# CellsFromSpace: a fast, accurate, and reference-free tool to deconvolve and annotate spatially distributed omics data

**DOI:** 10.1093/bioadv/vbae081

**Published:** 2024-05-30

**Authors:** Corentin Thuilliez, Gaël Moquin-Beaudry, Pierre Khneisser, Maria Eugenia Marques Da Costa, Slim Karkar, Hanane Boudhouche, Damien Drubay, Baptiste Audinot, Birgit Geoerger, Jean-Yves Scoazec, Nathalie Gaspar, Antonin Marchais

**Affiliations:** INSERM U1015, Gustave Roussy Cancer Campus, Université Paris-Saclay, Villejuif F-94805, France; INSERM U1015, Gustave Roussy Cancer Campus, Université Paris-Saclay, Villejuif F-94805, France; Department of Medical Biology and Pathology, Gustave Roussy Cancer Campus, Villejuif 94805, France; INSERM U1015, Gustave Roussy Cancer Campus, Université Paris-Saclay, Villejuif F-94805, France; Department of Pediatric and Adolescent Oncology, Gustave Roussy Cancer Campus, Université Paris-Saclay, Villejuif 94805, France; University Bordeaux, CNRS, IBGC, UMR, Bordeaux 33077, France; Bordeaux Bioinformatic Center CBiB, University of Bordeaux, Bordeaux 33000, France; INSERM U1015, Gustave Roussy Cancer Campus, Université Paris-Saclay, Villejuif F-94805, France; Office of Biostatistics and Epidemiology, Gustave Roussy, Université Paris-Saclay, Villejuif 94805, France; Inserm, Université Paris-Saclay, CESP U1018, Oncostat, Labeled Ligue Contre le Cancer, Villejuif 94805, France; INSERM U1015, Gustave Roussy Cancer Campus, Université Paris-Saclay, Villejuif F-94805, France; INSERM U1015, Gustave Roussy Cancer Campus, Université Paris-Saclay, Villejuif F-94805, France; Department of Pediatric and Adolescent Oncology, Gustave Roussy Cancer Campus, Université Paris-Saclay, Villejuif 94805, France; Department of Medical Biology and Pathology, Gustave Roussy Cancer Campus, Villejuif 94805, France; INSERM U1015, Gustave Roussy Cancer Campus, Université Paris-Saclay, Villejuif F-94805, France; Department of Pediatric and Adolescent Oncology, Gustave Roussy Cancer Campus, Université Paris-Saclay, Villejuif 94805, France; INSERM U1015, Gustave Roussy Cancer Campus, Université Paris-Saclay, Villejuif F-94805, France; Department of Pediatric and Adolescent Oncology, Gustave Roussy Cancer Campus, Université Paris-Saclay, Villejuif 94805, France

## Abstract

**Motivation:**

Spatial transcriptomics enables the analysis of cell crosstalk in healthy and diseased organs by capturing the transcriptomic profiles of millions of cells within their spatial contexts. However, spatial transcriptomics approaches also raise new computational challenges for the multidimensional data analysis associated with spatial coordinates.

**Results:**

In this context, we introduce a novel analytical framework called CellsFromSpace based on independent component analysis (ICA), which allows users to analyze various commercially available technologies without relying on a single-cell reference dataset. The ICA approach deployed in CellsFromSpace decomposes spatial transcriptomics data into interpretable components associated with distinct cell types or activities. ICA also enables noise or artifact reduction and subset analysis of cell types of interest through component selection. We demonstrate the flexibility and performance of CellsFromSpace using real-world samples to demonstrate ICA’s ability to successfully identify spatially distributed cells as well as rare diffuse cells, and quantitatively deconvolute datasets from the Visium, Slide-seq, MERSCOPE, and CosMX technologies. Comparative analysis with a current alternative reference-free deconvolution tool also highlights CellsFromSpace’s speed, scalability and accuracy in processing complex, even multisample datasets. CellsFromSpace also offers a user-friendly graphical interface enabling non-bioinformaticians to annotate and interpret components based on spatial distribution and contributor genes, and perform full downstream analysis.

**Availability and implementation:**

CellsFromSpace (CFS) is distributed as an R package available from github at https://github.com/gustaveroussy/CFS along with tutorials, examples, and detailed documentation.

## 1 Introduction

Spatial transcriptomics (ST) is a rapidly evolving field of powerful technologies enabling the analysis of spatial distribution and context of cell types and activities within tissues. Commercially available technologies are divided into two main categories: spatially barcoded next generation sequencing (NGS)-based [10X Visium (Ståhl *et al.* 2016), Slide-seqV2 now Curio Seeker (Rodriques *et al.* 2019), Stereo-seq (Chen *et al.* 2022)] and transcript level panel-based high-throughput imaging-based approaches, using either *in situ* hybridization [ISH-based, Vizgen MERSCOPE (Moffitt *et al.* 2016), Nanostring CosMX (He *et al.* 2022)] or *in situ* sequencing [ISS-based, 10x genomics Xenium (Ke *et al.* 2013)]. NGS-based approaches enable whole transcriptome analysis on tissue sections at varying degrees of resolution, from subcellular to quasi cellular resolution, depending on the technology. These methods generate highly dimensional, sparse, spatially distributed data, which can be computationally taxing, and still requires the development of new algorithms to jointly exploit the resulting molecular data, spatial coordinates, and tissue images (Bressan *et al.* 2023).

10X Genomic’s Visium technology, currently the most widespread commercial solution by publication metrics, outputs moderate size datasets at the cost of lower resolution compared to other techniques, with 55 µm diameter spots typically encompassing 1–20 cells. This cell mixture, however, results in spot-size “mini-bulks,” requiring alternative analytical approaches, as classical methods used in single cell analysis cluster spots primarily according to the cell mixture instead of biologically relevant phenotype. This is particularly problematic in tumor tissues, where cancer cells and their microenvironment are often diffuse and intermingled, complicating their respective analyses. Therefore, a deconvolution/decomposition step is often required for array-based NGS techniques to gain insight into the mixture of cells populating each spot. These deconvolution methods most often rely on single-cell RNA sequencing references (Andersson *et al.* 2020, Biancalani *et al.* 2021, Dong and Yuan 2021, Elosua-Bayes *et al.* 2021, Song and Su 2021, Cable *et al.* 2022, Danaher *et al.* 2022, Gayoso *et al.* 2022, Kleshchevnikov *et al.* 2022, Lopez *et al.* 2022, Li *et al.* 2023). This approach suffers from drawbacks such as the necessity for high-quality reference datasets, poor performance with cell identities absent from the reference, high computing requirements, low user-friendliness, and often a black-box processing.

Recently, Miller *et al.* (2022) proposed the reference-free deconvolution tool STdeconvolve to decompose ST signal using a Latent Dirichlet Allocation (LDA) method, typically a text processing algorithm, which approximates genes to words and spots to documents. Their work presents several interesting developments for the field of ST, but known drawbacks of LDA might limit its performance: sensitivity to noisy and sparse data, difficulty in detecting rare topics, difficulty in distinguishing similar topics (cell subtypes), fixed number of topics, preselection of highly variable abundant genes, and relatively high computational cost (Serdyukov 2013, Koltsov *et al.* 2014). Here, we propose a new reference-free decomposition framework for ST dataset, named CellsFromSpace (CFS) that overcomes most of the aforementioned limitations of LDA. CFS is based on independent component analysis (ICA), a blind source separation technique that attempts to extract signal sources from multiple source mixtures (Comon 1994, Hyvärinen 2013). ICA has been successfully applied to bulk transcriptomic data in hundreds of publications and shows the best performance over other methods to detect gene module from bulk RNA-seq (Saelens *et al.* 2018). ICA performance is directly dependent on the ratio between the number of sources and detectors. With thousands of spots (detectors) each covering a few cell types (sources), we hypothesized that ICA would be well suited for the decomposition of ST signals by cell of origin and distinct biological processes. Additionally, through the biological interpretation and spatial distribution analysis of the independent components (ICs), an expert in the field can supervise the removal of noise and artifacts, as is routinely performed in electroencephalography (EEG), functional magnetic resonance imaging (fMRI), and other recent advancements in neuroscience (McKeown *et al.* 2003, Schneider *et al.* 2023). Hence, in the ICA latent space of ST data, one can select, isolate, or remove background noise and parasitic signals, such as cell death or associated to histological artifact-associated (e.g. Bubbles), that are not relevant for downstream analysis, without altering the signal of interest. Finally, the permissiveness of ICA regarding over-decomposition (Sompairac *et al.* 2019) is a significant advantage that allows the fixation of an arbitrary large number *k* with a minimum drawback of generating near identical components, easily corrected by component annotations and subsequent merging. Altogether, the characteristics of ICA in combination with an expert-supervised annotation and component selection, as implemented in the CFS framework, enables: (i) to avoid dimensionality optimization steps, (ii) to select biologically relevant components and to remove noise, (iii) to study specific cell subtypes in the IC latent space, and (iv) to identify conserved signal though multi-sample ST datasets. Moreover, ST-derived positive IC weights can be converted into compositional values, allowing quantitative compositional analysis of spots.

When applied to ST analysis, CFS allows a biologically relevant and interpretable decomposition of the spot mixture into its subcomponents responsible for common expression patterns observed within the tissue, such as cell type signatures, biological processes, and tissue organization.

CFS consists of a preprocessing pipeline and an easy-to-use Shiny user interface (UI) to analyze, annotate, visualize, subset ST data, and more. CFS was used to fully process sample datasets from multiple major sequencing-based ST technologies and was also applied to ISH ST for performance and accuracy benchmarking. However, it proved to be a useful as an intermediary tool for sample screening and identification of regions of interest. This highlights the flexibility and effectiveness of a semisupervised ICA-based signal decomposition method for the analysis of ST in healthy and diseased samples. The companion shiny interface also enables non-bioinformaticians to quickly perform a complete analysis of ST data from Visium, Slide-seq, MERSCOPE, or CosMX dataset without prerequisite programing knowledge; and to generate *Seurat* (Hao *et al.* 2021) objects for subsequent downstream analyses.

## 2 Methods

### 2.1 CFS overview and use case

The package is split into two main components: (A) the package itself, which includes functions to preprocess data loaded in *Seurat* by (i) *prepare_data*, which normalizes the count matrix; (ii) *RunICA*, which runs the desired ICA algorithm (“icafast,” “icaimax,” “icajade”) to separate the signal into independent components (IC), corrects the signs of ICs (the side of the distribution with the highest absolute max value is attributed the positive sign), and filters out ICs with a kurtosis below a user-defined threshold (3 by default); (iii) *Enrich_ICA* which queries *EnrichR*(Chen *et al.* 2013, Kuleshov *et al.* 2016, Xie *et al.* 2021) databases to run and curate functional enrichment analyzes for individual ICs, and (iv) functions to convert ISH ST data into a format compatible with CFS, and (B) a Shiny UI to run the previously mentioned preprocessing steps and downstream annotation and analysis of ICA results. A critical step of CFS’s analytical workflow is the manual annotation of ICs by scientists. To improve the ease, speed, and efficacy of this critical step by biologists, clinicians, or other experts, CFS’s Shiny UI offers several visualization options such as: (i) global gene × IC heatmap of the top contributing genes for all ICs, (ii) spatial distribution of ICs and their contributing genes, (iii) IC-specific gene × IC and gene × spot heatmaps showing the contribution of IC-defining genes in ICs and cells, and (iv) annotated bar graph visualization of functional enrichment analyses from *EnrichR* of the contributor genes for each IC. *EnrichR* conducts over-representation analyses (ORA) on contributor gene lists to realize enrichment analysis on any of the tool’s 227 available libraries. By default, CFS queries libraries for cell type [*PanglaoDB* (Franzén *et al.* 2019), *Azimuth* (Hao *et al.* 2021), *Tabula sapiens* (The Tabula Sapiens Consortium *et al*. 2022) and *muris* (Tabula Muris Consortium *et al.* 2018)*, Cellmarker* (Zhang *et al.* 2019)] and functional [*MSigDB* (Liberzon *et al.* 2015)*, Gene Ontology* (Ashburner *et al.* 2000, Aleksander et al. 2023)*, KEGG* (Kanehisa 2019, Kanehisa and Goto 2000, Kanehisa *et al.* 2023), and *Reactome* (Jassal *et al.* 2019)] annotations.

Once annotated, relevant ICs can be used to calculate spot clustering and UMAP dimensionality reduction. Marker genes for the calculated clusters can be calculated and visualized within the Shiny UI. In addition, the interpretable ICA latent space can be used directly to investigate cellular dynamics within samples. To interpret cellular mixtures in the IC space of each spot, CFS provides spatial and UMAP scatter pie chart representations of data. These scatterpie representations allow the observation of all or a selected subset of ICs weight on each spot as well as an annotation-based categorical representation of ICs. Due to the assumptions of ICA, the absolute scaling between ICs is not meaningful. However, the relative weight of ICs within a spot can be used as a meaningful representation of cell type ratios and can be visualized using scatterpies. Density mapping of individual or combined IC weights can also be visualized by bivariate interpolation of proportional ratios of IC positive weights.

The Shiny UI allows for a complete analysis workflow from the loading of 10x Genomics *SpaceRanger* (v2.1) or any other processed ST output up to the easy exporting of publication-ready figures of all visualizations in png, jpeg, pdf, or svg format. Data generated within the application can also be exported in rds format integrally or subsetted from within the tool. The resulting *Seurat* objects containing all curated annotations, calculated metadata and dimensionality reductions can be loaded back into CFS for additional analyses or directly in R for more complex downstream analyses with compatible workflows.

A detailed description of CFS features and an in-depth tutorial of the standard CFS workflow are available on our github page (https://github.com/gustaveroussy/CFS).

### 2.2 Synthetic data

Synthetic data were generated using the package *synthspot* (v0.1.0: https://github.com/saeyslab/synthspot) (Sang-aram *et al.* 2024) using the cord blood mononucleated cell (cbmc) dataset (Stoeckius *et al.* 2017) obtained through the *SeuratData* package (v0.2.2.9001, https://github.com/satijalab/seurat-data). Mouse and multiplet cells were removed from the reference and monocytes and T lymphocyte subtypes were collapsed into a single annotation each. The *generate_synthetic_visium* function was used to generate spots containing 2 to 10 cells, forming 5 regions containing different ratios of distinct cell types with the “artificial diverse overlap” distribution. In total, 1672 spots were generated.

### 2.3 Spatial reference samples

The CellsFromSpace pipeline was applied to multiple real-life datasets: 10X Genomics Visium *Adult Mouse Brain (FFPE)*, Spatial Gene Expression Dataset by Space Ranger 1.3.0 (2021, August 16) (https://www.10xgenomics.com/datasets/adult-mouse-brain-ffpe-1-standard-1-3-0), *Human Breast Cancer: Ductal Carcinoma In Situ, Invasive Carcinoma (FFPE)*, Spatial Gene Expression Dataset by Space Ranger 1.3.0 (2021, June 9) (https://www.10xgenomics.com/datasets/human-breast-cancer-ductal-carcinoma-in-situ-invasive-carcinoma-ffpe-1-standard-1-3-0), and *Human Prostate Cancer, Adenocarcinoma with Invasive Carcinoma (FFPE),*Spatial Gene Expression Dataset by Space Ranger 1.3.0 (2021, June 9) (https://www.10xgenomics.com/datasets/human-prostate-cancer-adenocarcinoma-with-invasive-carcinoma-ffpe-1-standard-1-3-0), Slide-seqV2 mouse hippocampus sample dataset (ssHippo) (Stickels *et al.* 2021) was obtained through the *SeuratData* package (v0.2.2.9001), MERSCOPE sample dataset MERFISH Mouse Brain Receptor Map Slide 1 Replicate 1 was obtained from Vizgen (https://info.vizgen.com/mouse-brain-data), and CosMX sample datasets and annotations of formalin-fixed paraffin-embedded (FFPE) human non-small cell lung cancer were obtained from Nanostring (https://nanostring.com/products/cosmx-spatial-molecular-imager/ffpe-dataset/). *Seurat* v4.4.0 and v5.0.2 were used to analyze data.

### 2.4 Preprocessing pipeline

All samples were pre-processed using the CFS package and the following pipeline. Sample count matrices were first normalized using *Seurat’*s *sctransform* function. *Sctransform* uses regularized negative binomial regression to normalize single cell datasets (Hafemeister and Satija 2019), however, for ST data all genes were used as variables features and regression was realized on the number of counts by spots. Using the *ICASpatial* function, ICs were calculated for the ICA analysis with 600 iterations using the *Icafast* method. By default, 100 ICs are calculated to catch more independent signals than cell types expected in our samples at the risk of over decomposing signal since IC weights can later be recombined through addition. Only leptokurtic ICs (kurtosis > 3) are retained for downstream processing and analysis. By convention, IC sign correction is then applied to attribute a positive sign to the side of the distribution containing maximum absolute IC weights. The sign doesn't alter the interpretation of ICs, but we have empirically observed that this correction drastically improves the interpretation of ICs by experts and better fit with the biology. Functional enrichment analysis of IC-contributing genes (defined as genes with feature loading absolute *z*-score ≥ 3) is done using the *enrichR* package (v3.2) with the *Show_Enrich* function for the desired databases. To compare ICA performances in UMAP clustering calculation with other dimensional reductions methods, principal component analysis (PCA) and non-negative matrix factorization (NMF) were calculated using identical parameters.

### 2.5 Pseudotime analysis

Pseudotimes, branches, and diffusion maps were computed from the independent components associated with cancer, only for the spots containing significant cancer signal, using the *destiny* v2.0 package (Haghverdi *et al.* 2016). Briefly, destiny calculates a Diffusion Pseudo Time (DPT) from a Diffusion Map based on transition probability. Samples are then subsetted by branch and cross-validated glmnet is calculated against the pseudotime values using *cv.glmnet()* from the *glmnet* package (v4.1–8) (Tay *et al.* 2023) with the following parameters each time: alpha = 0.02, nfolds = 10, family = *Gaussian*, intercept = FALSE and nlamda = 500.

### 2.6 ISH data pseudospot binning

To process ISH technologies’ datasets within CFS, samples first needed to be converted into a pseudo-spot format. To do so, a count matrix was recreated using the reported detected transcript table from standard output using CFS’s *Create_vizgen_seurat* or *Create_CosMX_seurat* functions (detected_transcript.csv for MERSCOPE and tx_file.csv for CosMX) with transcripts placed in grids of variable bin sizes (40 × 40 µm for MERSCOPE, 200 × 200 px or 24 × 24 µm for CosMX). Each pseudo-spot of this grid was then treated similarly to a Visium spot with the corresponding associated transcript pseudo-counts. A *Seurat* object was then created using this matrix as the count matrix input, and the preprocessing pipeline was run as described previously. For the MERSCOPE sample, pseudospots with fewer than five total transcripts were filtered out.

### 2.7 ICA compositional conversion

For comparative performance assessment, the ICA weights obtained through CFS are converted to compositional values. To do so, we report the ratio of individual positive weights of sign-corrected ICs to total positive IC weights per spot. A high pass filter is then applied to these relative abundance values to eliminate noise.

### 2.8 Comparative performance with STDeconvolve

Synthetic data, Visium Mouse brain, breast cancer, and CosMX NSCLC datasets were deconvoluted using the standard STdeconvolve workflow. Briefly, all pseudospots previously filtered by feature count were processed. Genes used for the analysis were restricted using *restrictCorpus* function to restrict overdispersed genes. Genes above 5% and under 100% of pseudospots were included. Latent Dirichlet allocation (LDA) was applied to find *K* latent topics. For synthetic data, instead of using the standard *K* optimization approach, a *K* value of 30 was used to match for the number of IC generated using ICA. Topics were then automatically annotated and combined as described below. This approach yielded better performance than the standard optimized-*K* approach (data not shown) and thus was retained for accurate best-performance assessment.

For real-life samples, however, the standard *K*-optimization approach was: yielding 38 topics for Visium mouse brain, 30 for Visium breast cancer, 22 for Visium prostate cancer, and 57 for joint CosMX NSCLC. Spatial composition and gene signature correlations between STdeconvolve and CFS features were computed using Pearson’s *r* coefficient.

A comparative annotation performance assessment was conducted with the CosMX NSCLC datasets by extracting ground truth cell annotations from the Giotto-processed object of the NSCLC dataset and attributed by pseudospot. For error calculations in synthetic and CosMX datasets, ground truth cell types were mapped to ICs and topics by their gene signature, simulating perfect feature annotation. For this, each feature was annotated to the cell type with the highest Pearson’s *r* coefficient between feature gene weight and ground truth mean cell gene expression. Features with the same annotation were summed into the designated cell type. Relative cell type composition for each modality was calculated by pseudospot, with features contributing to <10% and 5%, respectively, for synthetic and CosMX datasets being filtered out for STdeconvolve and CFS. Isomeric log ratio transformation (ILR) (Egozcue 2003) was applied to the compositional data and root-mean-square error (RMSE) values calculated with,

$${\mathrm{RMSE}}_{A}=\;\sqrt{\frac{\sum {\left(y_{Ai}-{\hat{y}}_{Ai}\right)}^{2}}{N}}$$

where *N* is the number of pseudospots, y*Ai* the predicted proportion for ILR dimension A, and ŷ*Ai* the ground truth proportion for this dimension. One-tailed Diebold–Mariano test (Diebold and Mariano 1995) was used to compare overall RMSE between algorithms.

### 2.9 Comparative performance with Cell2location

Cell2location’s deconvolution workflow was also applied to the Synthetic data using two reference scRNAseq datasets: the original cbmc reference, constituting the “internal” reference, and the pbmc3k reference from 10X Genomics (https://support.10xgenomics.com/single-cell-gene-expression/datasets/1.1.0/pbmc3k) obtained through the *SeuratData* package, constituting the “external” reference and simulating the use of a slightly incomplete reference dataset, which is often the case for complex real-life samples such as cancerous tissues. In both cases, a detection alpha of 20 was employed as is classically used on Visium datasets. The mean prediction result was used and treated similarly to the prediction results from STdeconvolve and CFS. Features contributing to <10% were filtered out, ILR was applied, and RMSE values were calculated against ground truth values from the synthetic spot compositions.

## 3 Results

### 3.1 Visium reference data analysis

The efficiency of the CFS workflow was first assessed by analyzing the Spatial Gene Expression Visium datasets available from 10x Genomics. Visium fresh frozen and FFPE samples of adult mouse brain and human tumors were analyzed using our standard coding-free methodology directly in the Shiny application.

**Figure 1.**
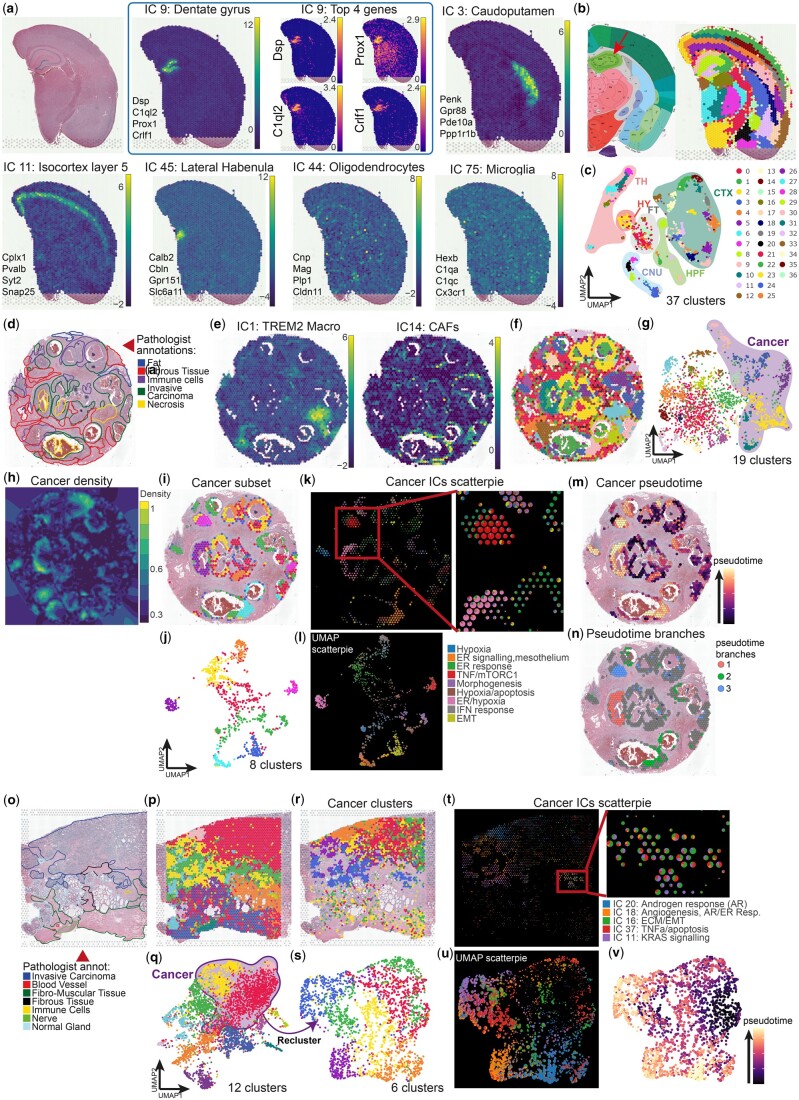
Analysis of Visium spatial transcriptomics data using CellsFromSpace’s independent component analysis pipeline. (a–c) Visium FFPE mouse brain analysis using CFS showing (a) H&E reference slide of the sample (top left) and examples of ICs associated to various brain substructures and diffuse cell types each with their top four contributor genes (See Supplementary Table S2 for full list of contributor genes), and spatial feature example of contributor genes for IC 9: Dentate gyrus. IC weights with color scale viridis, log-normalized counts for gene expression with color scale viridis plasma. (b) Reference slide from the Allen mouse brain Atlas (left) and identified substructures following spot clustering using Louvain algorithm at a 3.8 resolution with filtered and annotated ICs as input (right) showing excellent substructure resolution. Red arrow pointing at the pyramidal layer of dentate gyrus, darker green band within the hippocampal region. (c) Two-dimensional UMAP projection of spots after clustering with corresponding brain zones annotated. CNU: Cerebral nuclei, CTX: Cortex, DG: Dentate gyrus, FT: Fiber tracts, HPF: Hippocampus, HY: Hypothalamus, TH: Thalamus. Same cluster colors between panels (b-right) and (c) (d–n) Visium FFPE human breast cancer analysis using CFS. (d) Reference H&E slide with pathologist annotations from 10X Genomics. (e) Sample spatial projection of ICs related to distinct tumor stromal cells (See Supplementary Table S4 for full list of contributor genes). Spatial (f) and UMAP (g) projections of spot clustering using Louvain algorithm at a 1.2 resolution with all filtered and annotated ICs as input with cancer-associated clusters highlighted in the UMAP projection. (h) Kernel density projection on the spatial embedding of Cancer-associated signal (based on sum of cancer IC weights, see Supplementary Fig. S1 for tumor stroma components kernels) allowing for automatic subsetting of cancer-associated spots within CFS. Spatial (i) and UMAP (j) projections of cancer spots following manual subsetting within CFS colored after reclustering using Louvain algorithm at a 1.0 resolution with only cancer-associated ICs as input. Scatterpie representation of cancer IC weights in spatial (k) and UMAP (l) projections allows for the rapid visualization of the ICs associated with distinct spot clusters and their respective annotations within CFS. Spatial projection of pseudotime calculation (m) and distinct branches (n) following trajectory inference with Destiny’s DPT algorithm (see Supplementary Fig. S2) showing three clear cancer subpopulations within the sample. (o–v) Visium FFPE human prostate cancer analysis using CFS. (o) Reference H&E slide with pathologist annotations from 10X Genomics. Spatial (p) and UMAP (q) projections of spot clustering using Louvain algorithm at a 0.5 resolution with all filtered and annotated ICs as input with cancer-associated clusters highlighted in the UMAP projection. Spatial (r) and UMAP (s) projections of cancer spots following manual subsetting within CFS colored after reclustering using Louvain algorithm at a 0.5 resolution with only cancer-associated ICs as input. Scatterpie representation of cancer IC weights in spatial (t) and UMAP (u) projections allows for the rapid visualization of the ICs associated with distinct spot clusters and their respective annotations within CFS. UMAP projection of pseudotime calculation (v) following trajectory inference with Destiny’s DPT algorithm (see Supplementary Fig. S3) showing cancer subpopulations within the sample. Cluster colors for all panels follow the legend present in (c) for the number of clusters specified in the UMAP panel.

For the FFPE mouse brain sample, after standard preprocessing with CFS (see Section 2), 92 ICs passed the kurtosis threshold for downstream analysis. Taking advantage of the annotation tools included in the Shiny app, in half a day, 75 ICs were manually annotated by a biologist as relevant based on their distribution pattern or gene signature (Supplementary Table S1). Most ICs were directly associated with well-defined brain structures, demonstrating the direct capture of spatially distributed gene co-expression by ICs. Interestingly, ICA also captured ICs associated with diffuse or infiltrating cell populations such as microglia or oligodendrocytes (Fig. 1a) after manual curation of functional enrichments of contributor genes (Supplementary Table S2). This confirms the capacity of ICA to isolate cell type specific signal to enable reference-free signal decomposition. Then, from the Shiny app, spot clustering using a Louvain algorithm (resolution = 3.8) based on these 75 dimensions generated 37 clusters that closely recapitulated the layers and substructures of the mouse brain compared with the Allen mouse brain atlas reference for the same coronal layer (position 269, Fig. 1b). For instance, cortical layers or Ammon’s horn pyramidal layer sections are clearly defined, within the limits of Visium’s spot resolution, as distinct clusters because most layers are explained by specific ICs. The distribution of clustered spot in the UMAP embedding (Fig. 1c) demonstrated the unambiguous spot cluster assignment emphasizing the benefits of the expert annotation and the exclusion of ICs considered as noise or specific to one spot (over-decomposition).

Efficient for the healthy brain, a well-regionalized organ, CFS’ ability to capture both regionalized and isolated cell signatures is well suited for medical research projects, where characterization of small groups of cells exhibiting distinct behaviors is essential. This is of particular interest for the analysis of spatially heterogeneous tissues with less defined strata and more invasive cells such as tumors. We, thus, analyzed the Visium FFPE human breast (Fig. 1d and Supplementary Tables S3 and S4) and prostate (Fig. 1o and Supplementary Tables S5 and S6) cancer samples from 10X Genomics to determine the cell type composition and biological activities within spots of tumor tissues. After IC annotation and filtering, 46 ICs (Fig. 1e–g and Supplementary Table S3) and 36 ICs (Fig. 1p and q), were used for spot clustering of breast and prostate cancers, respectively, yielding clusters closely mapping to the pathologist annotations for each tissue. Because of the size of Visium spots, most are composed of multiple cell types in such tissues. Two-dimensional embedding and clustering thus tends to be driven by cellular mixture (i.e. neighborhood) rather than real identity. However, using the interpretable ICA latent space, distinct cell types can be directly mapped both spatially and onto the UMAP embedding. CFS also allows kernel density mapping of IC categories to easily visualize the distribution of signal associated with broad cell types of interest, such as cancer cells, lymphoid, myeloid, or stromal cells within tumors (Fig. 1h and Supplementary Fig. S1). This constitutes a semi-supervised strategy implemented within CFS to subset spots of interest with specific annotation. Alternatively, spot subsetting can be performed manually based on cluster identity following clustering, for instance, to isolate spots with a cancer signature based on IC enrichment (Fig. 1i and r). Spot reclustering of the subsetted object using only cancer-associated ICs allows for a finer dissection of cancer phenotypes within samples (Fig. 1j and s). To analyze further, CFS integrates multiple visualization tools to interrogate the cellular composition and distribution within samples. For instance, scatter pie representation allows for visual breakdown of cellular composition for cell populations based on IC annotation both in spatial (Fig. 1k and t) and UMAP (Fig. 1l and u) embeddings, allowing the interpretation of relevant pathways and analysis of component relationships. The isolation of spots and ICs specific to cell types and their export in a Seurat object (directly from the Shiny app) enables downstream analysis using any other compatible packages. For instance, a trajectory inference analysis of the breast cancerous spots was done using the Destiny package directly on the IC latent space (Fig. 1m and Supplementary Fig. S2a) and revealed 3 distinct phenotypic branches (Fig. 1n and Supplementary Fig. S2b and c). Extraction of genes associated with each branch using *glmnet* (alpha 0.02, Supplementary Fig. S2d and e) revealed three subpopulations characterized by (1) high IGFBP5, GSTP1, and GNAT3 and low SERF2 expression, (2) high TGM2, SERPINA3, IL32, UBD, and ICAM1 and low SCGB1D2, SCGB2A2, AZGP1, MUCL1, and DBI expression, and (3) high SOD2 and low SCGB1D2, AZGP1, and DBI expression.

For the prostate cancer sample, the same trajectory inference methodology (Fig. 1u and Supplementary Fig. S3a) also identified three branches (Supplementary Fig. S3b–d), although less defined, and a *glmnet* analysis revealed the genes associated with these phenotypic paths and distinguished them by their expression of ODC1, SPON2, ADGRF1, TSPAN8, CLDN3, and KRT8 among others (Supplementary Fig. S3e and f).

Using ICA, CFS efficiently identified distinct cancerous cell phenotypes within a tumor. Furthermore, it identified and distinguished stromal signatures such as immune infiltrates without the use of prior knowledge about the sample or reliance on external reference single cell datasets, which is particularly relevant for highly heterogeneous tissues such as cancer, where reference atlases might not faithfully recapitulate the patient’s cancer phenotype or tumor composition.

### 3.2 Slide-seqV2 data analysis

**Figure 2.**
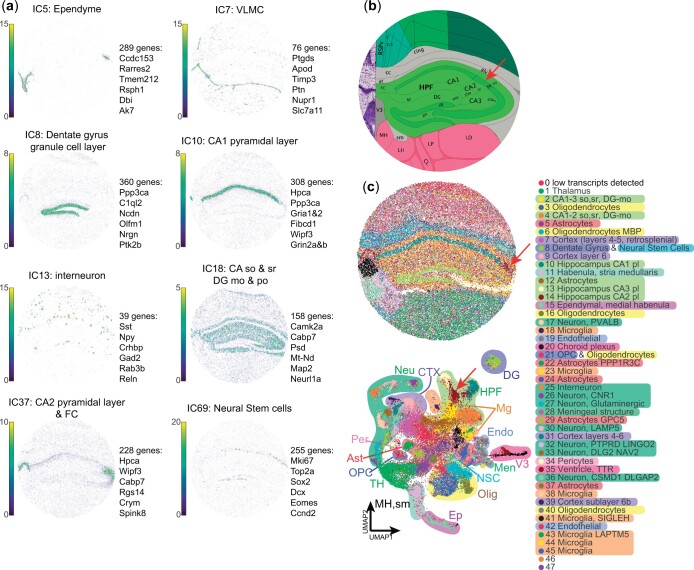
High resolution Slide-seqV2analysis using CFS allows for fine substructure definition and signal deconvolution. (a) Sample spatial projections of substructure- or cell type-associated ICs colored by weight with their 6 most contributor genes (see Supplementary Table S8 for full list of gene contribution by IC and Supplementary Table S7 for IC annotations). (b) Allen brain atlas reference for region of interest. (c) Spatial (top) and UMAP (bottom) projection of spots clustered using Louvain algorithm at a 0.95 resolution using all filtered and annotated ICs as input with detailed and broad (shading) cluster annotations. Red arrows in (b) and (c) point at the CA2 pyramidal layer. Ast: Astrocytes, CA: Ammon’s horn, CTX: Cortex, DG: Dentate gyrus, Endo: Endothelial, Ep: Ependyme, FC: Fasciola cinerea, HPF: Hippocampal formation, Men: Meningeal substructure, Mg: Microglia, MH: Medial habenula, mo: Molecular layer, Neu: Neuron, NSC: Neural stem cell, Olig: Oligodendrocyte, OPC: Oligodendrocyte progenitor cell, Per: Pericyte, po: Polymorph layer, sm: Stria medullaris, so: Stria oriens, sr: Stria radiatum, TH: Thalamus, V3: 3rd Ventricle, VLMC: vascular and leptomeningeal cell.

To demonstrate the compatibility of CFS with other technologies, we analyzed the *ssHippo* mouse hippocampus Slide-seqV2 reference data from the *SeuratData* package using CFS’s standard pipeline. Despite Slide-seqV2 enabling near-single cell resolution (10 µm diameter spots), the spatial array capture methodology (here using microbeads) still leads to the capture of cell mixtures. Therefore, we expect ICA to remain very efficient in identifying structure- and cell type-specific signals. Of the 100 computed ICs, none were threshold out by kurtosis value, and 70 were retained after manual annotation (Supplementary Tables S7 and S8). Thirty ICs were removed after thorough annotation by a biologist because the signal was not biologically meaningful (considered as “noise”), or was explained by only one or few spots. The remaining ICs were found to be associated with both (A) brain ontologies (ex. IC 8: dentate gyrus granule cell layer, IC 10: Ammon’s horn field 1 pyramidal layer, IC 18: Hippocampal stratum oriens & radiatum and molecular and polymorph layers of the dentate gyrus, IC 37: Ammon’s horn field 2 pyramidal layer and Fasciola cinerea, Fig. 2a), and (B) distinct cell types (ex. IC 5: ependymal cells, IC 7: ventricular and leptomeningeal cells (VLMC), 7 varieties of neurons, including IC 13: interneurons, and IC 69: proliferating neural stem cells, Fig. 2a). Spot clustering based on these ICs allowed for detailed mapping of the mouse hippocampal region with a resolution comparable to that of the Allen mouse brain atlas reference (Fig. 2b). Indeed, of the 48 recovered clusters obtained with Louvain resolution of 0.95, 45 could be directly annotated based on IC representation (Fig. 2c). Of note, using this approach, we were able to successfully identify a cluster of spots (Fig. 2c; cluster 14) associated with the small CA2 pyramidal layer (pl), which is typically missed by other deconvolution and clustering algorithms (Ma and Zhou 2022, Shang and Zhou 2022, Long *et al.* 2023, Singhal *et al.* 2024). Differential expression of CA2pl spots in comparison with CA1pl and CA3pl (clusters 10 and 13, respectively) revealed lower expression levels of calcium channel-related genes such as calmodulin 2 (*Calm2*), ATPase plasma membrane Ca2+ transporting 1 (*Atp2b1*), Protein phosphatase 3 catalytic subunit alpha (*Ppp3ca*), neurogranin (*Nrgn*), protein kinase C beta (*Prkcb*), and an increase in Purkinje cell protein 4 (*Pcp4*) a modulator of calmodulin activity.

Interestingly, distinct clusters were found to be associated with either brain ontologies such as the dentate gyrus, third ventricle, or thalamus or to more diffuse cell populations like neurons, microglia, oligodendrocytes, or astrocytes. Multiple subclusters are often identified for these diffuse populations with specific transcriptional signatures. For instance, 11 ICs were annotated as Neuronal with distinct gene signatures and distribution patterns (Supplementary Table S7). These results underline the complexity of biological tissues, even those as organized as the brain where biologically relevant infiltrating cells are abundant, and the capacity of ICA to finely detect such cells based on their specific transcriptomic signature, without prior knowledge.

### 3.3 ISH-based ST data analysis

Taking advantage of the continuous transcript capture performed by the Vizgen MERSCOPE and NanoString CosMX technologies, we assessed the scalability and robustness of CFS to varying spot resolutions and a low number of capture features.

**Figure 3.**
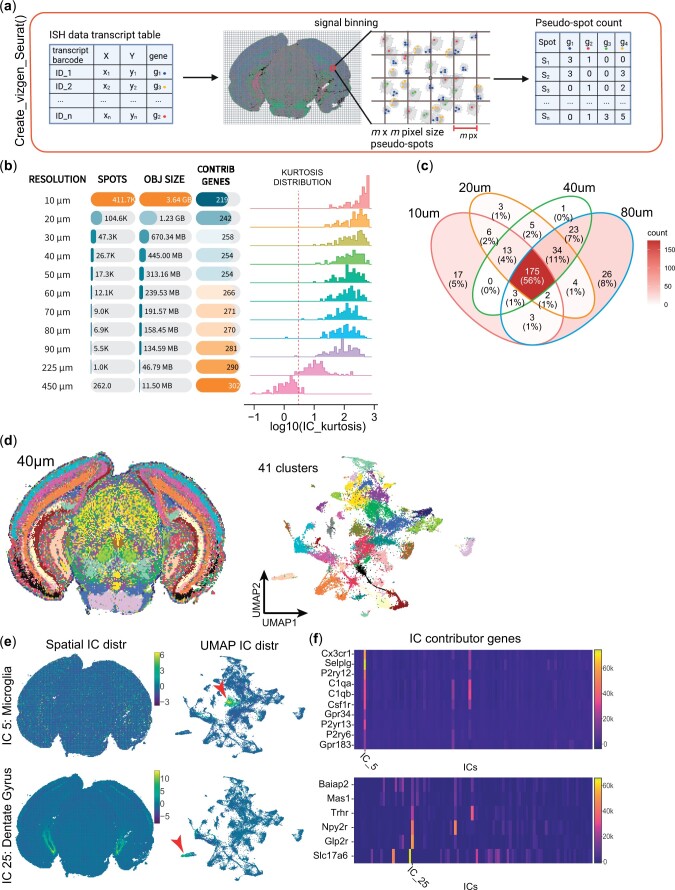
CFS allows for the analysis of ISH-based spatial transcriptomics data at varying degrees of resolution. (a) Schematic of the methodology behind the “*Create_vizgen_Seurat()*” function of CFS which generates pseudospot of user-defined *m* × *m* pixel size containing transcripts tabulated by the MERSCOPE technology to generate a count matrix input for the initiation of a Seurat object to input into the CFS Shiny application. (b) Recapitulative table of the impact of bin size *m* on object size, number of total contributor genes and kurtosis distribution of ICs (See Supplementary Fig. S4 for spatial and UMAP projections at different resolutions). (c) Venn diagram of contributor gene showing that 92% or more of contributor genes are detected in at least 2 levels of resolution with 225/254 (88.6%) contributor genes at *m* = 40 µm found in 3 or all 4 resolution levels (bold). (d–e) Sample analysis using CFS’s Shiny application for ICA signal deconvolution and annotation. (d) Spatial (left) and UMAP (right) projection of 40 × 40 µm pseudospots clustered using Louvain algorithm at a 1.0 resolution using all filtered and annotated ICs as input, generating 41 distinct pseudospot clusters. (e) Examples of spatial (left) and UMAP (center) projections of ICs associated to microglia (IC 5, top) and dentate gyrus substructure (IC 25, bottom) showing their distinct localization on the UMAP space, suggesting this level of resolution is sufficient to limit cell mixtures within pseudospots and capture specific cell populations. (f) IC feature loadings value heatmap of contributor genes associated to the ICs in (e) from the 438 genes probed in the MERSCOPE experiment.

To do so, we included methods to process ISH-based ST within CFS, such as user-defined pseudo-spot generation functions to generate CFS-compatible inputs from standard MERSCOPE and CosMX outputs. This step was conducted as described in the Methods section and is illustrated in Fig. 3a. When studying the impact of pseudospot size on ICA outcome (Fig. 3b), IC kurtosis distribution, used as a descriptor of IC super Gaussianity, was found to decrease as pseudo-spot size increased. Interestingly, the total number of IC-defining genes was observed to increase with pseudo-spot sizes, ranging from 219 to 302 unique contributing genes, in 10 and 450 µm bin sizes, respectively, out of the 438 probed genes for this dataset (Fig. 3b), with most being found at multiple resolution levels (Fig. 3c). Smaller bin sizes increased the number and resolution of pseudospot clusters (Supplementary Fig. S4). Most bin sizes between 20 and 90 µm appeared to be usable for this dataset. However, the 10 µm resolution appeared to constitute a breakpoint for ICA in terms of clustering resolution and interpretability in addition to being computationally intensive. This might be due to the relative sparsity of cells and transcripts in the analyzed tissue, leading to most pseudospots at the 10 µm resolution to contain less than a cell on average (78 329 cells for 411.7k spots). We also observed 29 ICs having a single contributor gene (versus 10 at 20 µm and 6 at 40 µm) and 57 being explained by three or fewer genes, suggesting that ICA captures more distinct gene expression patterns than complex signatures at this resolution with low number of features. We thus suppose that this breakpoint resolution is likely to be mostly dependent on the number of features and capture efficiency of the technology.

Despite the relatively low number of genes probed in ISH-based technologies compared to whole transcriptome sequencing approaches, CFS still allowed the isolation of structure- and cell type-specific signals. For instance, a more detailed analysis was performed with the 40 µm bin size MERSCOPE brain dataset composed of 438 genes, yielding 100 leptokurtic ICs (min 12,78, max 588.60, Fig. 3d and Supplementary Tables S9 and S10), of which manual curation retained 63 with highly specific signals for cell types [IC 4: astrocytes, IC 5: microglia (Fig. 3e top), IC 14: endothelial cells] and brain ontologies [IC 8: pons, IC 25: dentate gyrus granule cell layer (Fig. 3e bottom), IC 87: cerebral aqueduct (Supplementary Table S9)]. Thus, the use of a limited set of targeted genes in ISH-based methods as opposed to whole transcriptome does not appear to impair the ability of ICA to identify cell- and ontology-specific signals (Supplementary Table S10). However, the interpretation of ICs can be more imprecise based on the limited number of contributor genes (Fig. 3f).

The distribution of publicly available cancer datasets from the CosMX SMI platform by NanoString also allowed for the evaluation of the modified CFS pipeline on FISH-based nonstructured tumor tissue to assess the ability of ICA to deconvolute spatial signals of a more restricted dimensional nature (980 probed genes). The CosMX NSCLC dataset comprises multiple samples from distinct presentations of NSCLC from different donors (5 donors, 8 samples), allowing for joint sample analysis using the CFS workflow. Simply, samples were first binned as described previously (Fig. 3a, Methods) using the *Create_CosMX_seurat* function with a 200 × 200 px (24 × 24 µm) pseudospot resolution. All eight Seurat objects were then merged into a single 114 724 pseudospot object and processed simultaneously with the preprocessing pipeline (see Section 2). All 100 ICs obtained passed the kurtosis threshold and 81 were kept following manual annotation (Supplementary Tables S11 and S12). Of the 980 genes assayed, 355 (36.2%) were identified as IC-defining contributor genes. The filtered ICs describe common parenchymal and immune signatures for the tumor stroma, and common tumor programs between samples while uncovering phenotypical variations between tumors, as described by sample-enriched ICs (Supplementary Fig. S5).

**Figure 4.**
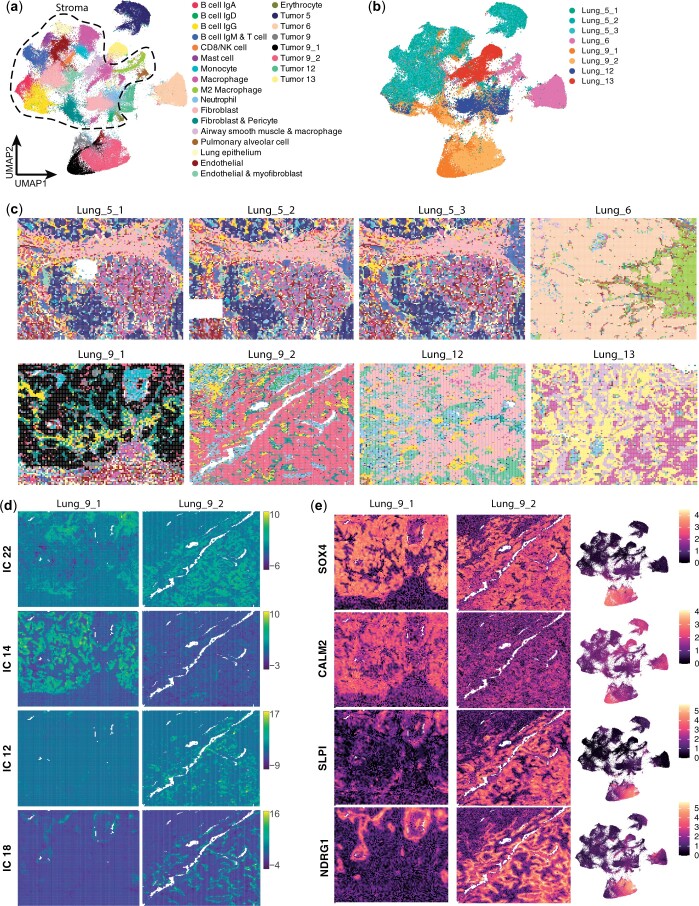
CFS enables the joint analysis of multiple datasets to identify common and sample or patient specific gene signatures. After applying pseudospot generation methodology and sample merging of the Nanostring CosMX Lung cancer dataset, the combined object was analyzed using the standard CFS pipeline. (a) UMAP projection of 24 × 24 µm pseudospot clustered using Louvain algorithm at a 1.0 resolution and annotated using top contributor ICs. Dotted line highlights pseudospots composed mostly of stromal components. (b) Coloring of pseudospots on the UMAP embedding from (a) based on sample of origin, showing how certain clusters are shared between samples. (c) Spatial distribution of pseudospot clusters calculated in (a) showing the relative composition in shared (i.e. B cells IgG: yellow, Macrophages: magenta, OLFM4 multiple tumor: light blue grey) versus sample-specific clusters, notably in both samples of patient 9 (Lung_9_1 & Lung_9_2, bottom left). (d, e) Detailed interrogation of common and distinct transcriptomic signatures between samples of patient 9. (d) Spatial projection of 4/9 Lung_9 cancer-associated ICs (see Supplementary Table S11 for all IC annotations) on sample Lung_9_1 (left) and Lung_9_2 (right). (e) Spatial (Lung_9_1: left, Lung_9_2: center) and UMAP (right) projections of top common (SOX4, top) and sample-specific genes (CALM2, SLPI, NDRG1, bottom 3) as determined by IC contribution and differential gene expression analysis between clusters defined in (a).

Pseudospot clustering and annotation allowed the identification of multiple immune and stromal populations along with cancer-specific clusters (Fig. 4a). Although most cancer clusters were found to be patient-specific, immune and stromal clusters were found in more than one patient, such as IgD and IgG-secreting B cells, macrophages, fibroblasts, and erythrocytes (Fig. 4b and Supplementary Fig. S6). Of note, a neutrophil population with high expression of olfactomedin 4 (OLFM4) was found to be present in all samples. Spatial projection of these clusters (Fig. 4c) allows for the rapid identification of these shared (or distinct) populations of interest for further analyses at a transcript level with other software solutions.

Interestingly, CFS demonstrated ability to characterize intra and inter patient tumor heterogeneity when performing multi-sample joint analysis: While samples from different patients were well distinguished in the UMAP projection, cancer-associated pseudospots from both samples from patient 9, taken in different parts of the same tumor, clustered closely while remaining locally distinguishable (Fig. 4a, black and muted red clusters), allowing for the interrogation of the intratumoral heterogeneity in a spatially resolved manner. Both tumor sections show IgG B cell infiltration and fibroblast & pericyte stromal components (Fig. 4c yellow and dark teal clusters). Using ICA, we identified nine ICs associated with the cancer component of these tumors, some shared between both samples (ex. IC 22) and others specific to one or the other (ex. ICs 12, 14, and 18, Fig. 4d). Analysis of contributor genes to these ICs (Fig. 4e) revealed that SRY-box transcription factor 4 (*SOX4*) is expressed exclusively in patient 9 by all cancer cells, but sample 9_1 is characterized notably by the expression of calmodulin 2 (*CALM2*) whereas sample 9_2 cancer cells are enriched in secretory leukocyte peptidase inhibitor (*SLPI*) and N-myc downstream regulated 1 (*NDRG1*).

### 3.4 CFS reference-free deconvolution performance assessment

Reference-free unsupervised signal deconvolution is becoming an important aspect of ST analysis due to the limitations of single-cell reference-based approaches. Some of these limitations include the lack of relevant high quality single cell atlases for pathologic conditions or difficult-to-process tissues, or cell capture bias leading to missing cellular identities. The performance of supervised approaches is also strongly dependent on the quality of the reference’s annotation. At the time of writing, according to the benchmark by Li *et al.* (2023), the best published tool for reference-free deconvolution is STdeconvolve, which uses an LDA modeling approach for an optimized *K* number of topics for each dataset. We, thus, used it as a reference to evaluate CFS’s signal deconvolution performance.

**Figure 5.**
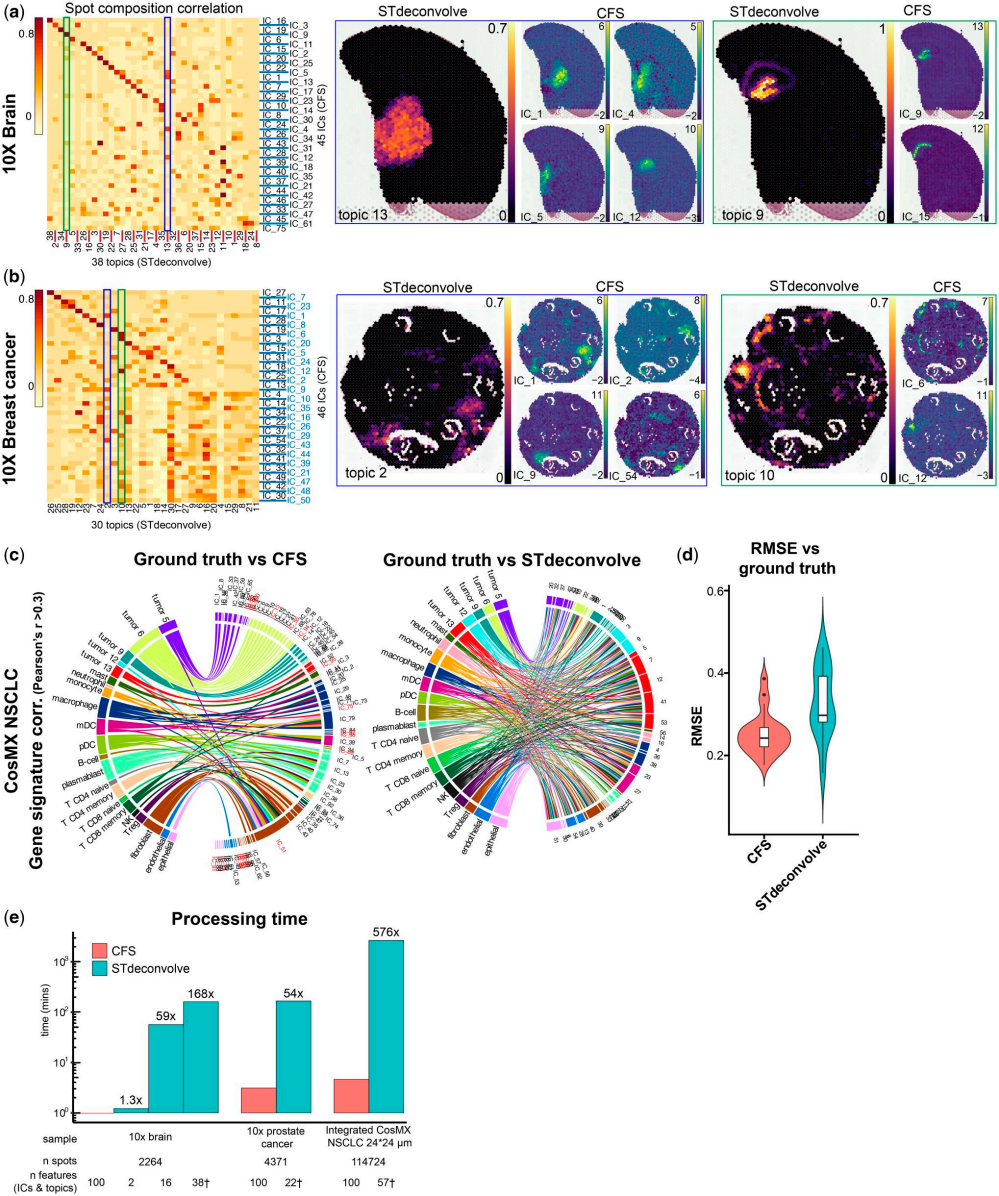
Comparative analysis of reference-free deconvolution results from CFS and STdeconvolve. (a, b) Correlation analysis based on spot composition of reference-free deconvolution tools CFS and STdeconvolve (left) with two highlighted examples (blue and green outlines) of STdeconvolve topics vs CFS ICs for a specific structure in the 10X brain (a) and 10X breast cancer (b) samples. See Supplementary Fig. S7 for more examples. Values represented as compositional (STdeconvolve) or IC weight (CFS) (c) Gene signature signal correlation of CFS ICs and STdeconvolve topics vs. ground truth (left and right, respectively) displaying all Pearson’s correlations *r* values > 0.3. IC names in red indicate ICs that were rejected by manual annotation (Fig. 4), but were kept for unsupervised performance assessment, note IC_51 with high correlation with most cell types. See Supplementary Fig. S8 for pseudospot compositional correlations (d) Root-mean-square-error (RMSE) of the deconvolved compositions using CFS or STdeconvolve (*n *=* *112 822 pseudospots) compared to ground truth after ILR-transformation. One-tailed Diebold–Mariano *P* value 1.473 × 10^−5^. (e) Processing time comparison between CFS (red) and STdeconvolve (teal) shows the exponential benefit of CFS in processing time with increased dataset size. “*n* Features represents” the number of user-defined features (ICs for CFS, topics for STdeconvolve) for each processing run, with * indicating the optimal *K* value defined by STdeconvolve for this sample.

On the highly structured mouse brain sample from 10X Visium, we obtained 45 high quality ICs using the CFS pipeline while STdeconvolve found the *K* = 38 condition to be optimal. All STdeconvolve topics were recapitulated by one or more ICs both by spatial distribution and contributor gene weight (Fig. 5a left and Supplementary Fig. S7a left). In most cases, such as in topics 13 or 9 (Fig. 5a blue and green outlines, respectively), CFS was able to further decompose the signal into 4 and 2 major components, often with a higher spatial definition (Supplementary Fig. S7b). Similarly, for the heterogeneous 10X Visium breast cancer dataset, CFS yielded 46 ICs to STdeconvolve’s 30 topics (Fig. 5b left), with CFS components often appearing more spatially defined (Fig. 5b blue and green outlines and Supplementary Fig. S7c).

## 4 Discussion

In this article, we presented CellsFromSpace or CFS, a user-friendly and reference-free analytical framework for spatial transcriptomics data. By leveraging independent component analysis, CFS is able extracts both spatially defined and diffuse cell signatures to deconvolute and jointly analyze multi-sample ST datasets.

To capture all of the biological complexity of the analyzed samples, we have created the CFS pipeline to favor lenient parameters coupled with assisted manual curation of the data. To avoid rare gene dropouts, especially in multi-sample joint analysis, and because of the high processing efficiency of ICA, CFS defaults to using all genes as input features with no discernable negative impact (Supplementary Fig. S9). As mentioned previously, we favor the over-decomposition of data by computing a high number of ICs (100 by default). Assisted manual curation of results allows for user-defined merging of ICs if they appear over-decomposed, but also eliminating non-biologically relevant signal. ICA being able to capture signal sources shared by multiple cell types (response to hypoxia, cellular division, etc.), selecting a number of components based on number of expected cell types can lead to under-decomposition (Supplementary Fig. S10), which is problematic.

The cornerstone of the CFS framework is the manual curation and annotation of ICs generated by the method. While time consuming, this critical step not only allows for the elimination of non-specific noise, but also consists of the main data interpretation step. To facilitate and enhance this crucial step, we created an easy-to-use Shiny UI to provide any user (clinician, biologist, bioinformatician) with all the analytical and visualization tools to easily and confidently annotate and interpret the data generated with major ST technologies currently commercially available. Manual IC annotation allows for the identification of cell types and states within samples and, among other things, the identification of genes with similar spatial distributions, defined as IC contributor genes. Manual curation also allows for the elimination of nonspecific, over-specific, redundant, artifactual, or uninterpretable ICs, thus, denoising the dataset with a minimal loss of relevant signals thanks to the independent property of ICA. Manual curation also has the added benefit of countering the ICA’s robustness shortcomings. Indeed, while exact feature loadings values might differ between iterations (Supplementary Figs S9 and S10), our experience shows that contributor gene lists remain sufficiently similar to be consistently interpreted, leading to all annotations being maintained between repeat experiments.

While we believe in remaining in the latent ICA space for data interpretation and analysis, CFS also implements the traditional workflow used in single cell transcriptomics and ST, which collapses the latent dimensions into clusters and two-dimensional projections for easy data visualization (UMAP, t-SNE, etc.) and downstream differential gene expression analysis of clusters.

ICA has seen a steady rise in popularity in the last few decades in a wide variety of application fields, in many cases ushering in algorithmic improvements to adapt the computational method to data type specificity. While ICA has successfully been used for some time for the analysis of various omics data (Sompairac *et al.* 2019), ST appears as a new field where ICA is natively well adapted when compared to other dimensionality reduction methods such as PCA or other blind source separation (BSS) algorithms such as NMF (Supplementary Fig. S11a–c) beyond previously known limitations of performance and interpretability (Mirzal 2017, Sompairac *et al.* 2019). In its simplest form, ICA also performs as well as or better than more elaborate techniques such as LDA or reference-based Bayesian modeling in deconvolution tasks (Fig. 5d and Supplementary Fig. S11d and e) at a fraction of the computational cost. Our application of ICA in the context of ST also revealed particularly useful properties for cellular biology, namely (1) the importance of signal sign, where only one tail of the IC distribution is usually associated to an interpretable cellular source, and (2) The surprising accuracy of positive ICs weights ratios for cellular abundance prediction within sources (spots) for direct proportional deconvolution of signal (Fig. 5d and Supplementary Fig. S11d and e). The latter enhances ICA’s performance as a reference-free signal deconvolution approach. By applying IC abundance high-pass filters, we observed significant reduction in prediction noise and increase in accuracy, as demonstrated by lower RMSE values (Supplementary Fig. S12). Higher filter values, however, led to cell type and spot dropouts, highlighting the necessity of proper adjustment of this parameter to the dataset.

Unsupervised reference-free signal deconvolution methods present considerable advantages over reference-based methodologies relying on single cell atlases. Beyond independence from the availability of high-quality reference datasets, reference-free methods are unaffected by single-cell methods’ limitations, such as compositional or transcriptional processing artifacts (Denisenko *et al.* 2020, Marsh *et al.* 2022) or the quality of single-cell atlas annotations, which can propagate cell misclassifications. Reference-free approaches are also useful for conditions for which single cell atlases might not be able to recapitulate unique phenotypes, such as cancer cells with high interpatient heterogeneity. Finally, by avoiding the identification and processing of a high-quality reference dataset, sample processing is made simpler and more accessible.

Reference-free techniques have previously been shown to perform well on certain real-world data, especially Visium samples (Li *et al.* 2023). Performance assessment showing that CFS outperforms STdeconvolve with a higher specificity and log-scale acceleration of the processing time validates the relevance of ICA and the CFS framework in ST data deconvolution and analysis. By aiming at minimizing assumptions regarding the extracted components, as they do not always represent cell types but sometimes describe cell activities or cell-to-cell interactions, CFS enables a deeper analysis of tissue- and cell dynamics within samples. This approach allows users who are experts in their respective fields the freedom to concentrate their ST data analysis on the signal they consider relevant.

Further development of CFS is underway for methodological improvements and enhanced downstream analysis. First, more exotic flavors of ICA such as non-negative ICA (Plumbley 2003) are being evaluated and adapted to further improve the performance and robustness of the method. IC interplay analysis is also being developed. Cross-correlation of 2D signal remains an unresolved area of data analysis with great potential for ST experiments in order, for instance, to identify co-localizing, mutually exclusive, or interfacing signals. For example, such analyses would be of great value to map cellular interactions between cancer and immune effector cells to identify potential mechanisms of tumor rejection or evasion and identify key molecular drivers of these interactions via ligand–receptor analyses.

In this work, we presented a new framework and tool for the analysis of spatial transcriptomics data. CellsFromSpace is versatile with its support for all commercially available ST technologies, is independent of high-quality reference datasets, is easy to use with its Shiny UI, is compatible with other single cell and ST analysis packages, and easily allows for the joint analysis of multiple samples. We hope that CFS will increase the accessibility and ease of ST data analysis to researchers and improve data interpretation.

## Supplementary Material

vbae081_Supplementary_Data

## Data Availability

CFS is an R package which can be downloaded in R using the *devtools* package from github directly at https://github.com/gustaveroussy/CFS along with tutorials, examples and detailed documentation.
