# Supplementary material for: CellsFromSpace: a fast, accurate, and reference-free tool to deconvolve and annotate spatially distributed omics data
Source: Bioinform Adv. 2024 May 30;4(1):vbae081. doi: 10.1093/bioadv/vbae081 (PMC11194756; doi:10.1093/bioadv/vbae081)
Supplement: vbae081_Supplementary_Data [file vbae081_supplementary_data.zip › SuppFigure5_dotplotCosMX_Censored.pdf]

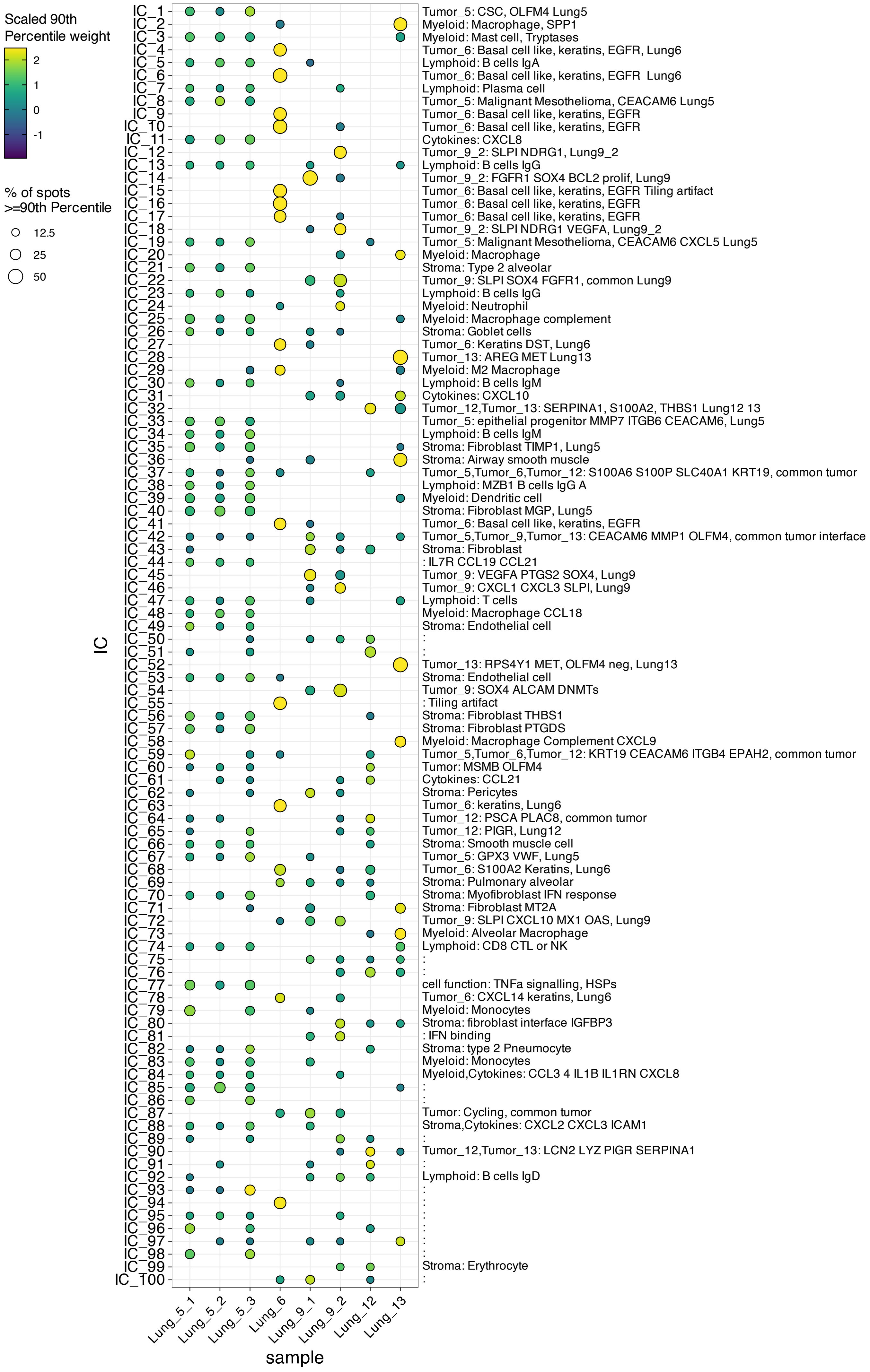
Scaled 90th Percentile weight
2
1
0
-1
% of spots >=90th Percentile
12.5
25
50
IC
IC_1 Tumor_5: CSC, OLFM4 Lung5
IC_2 Myeloid: Macrophage, SPP1
IC_3 Myeloid: Mast cell, Tryptases
IC_4 Tumor_6: Basal cell like, keratins, EGFR, Lung6
IC_5 Lymphoid: B cells IgA
IC_6 Tumor_6: Basal cell like, keratins, EGFR Lung6
IC_7 Lymphoid: Plasma cell
IC_8 Tumor_5: Malignant Mesothelioma, CEACAM6 Lung5
IC_9 Tumor_6: Basal cell like, keratins, EGFR
IC_10 Tumor_6: Basal cell like, keratins, EGFR
IC_11 Cytokines: CXCL8
IC_12 Tumor_9_2: SLPI NDRG1, Lung9_2
IC_13 Lymphoid: B cells IgG
IC_14 Tumor_9_2: FGFR1 SOX4 BCL2 prolif, Lung9
IC_15 Tumor_6: Basal cell like, keratins, EGFR Tiling artifact
IC_16 Tumor_6: Basal cell like, keratins, EGFR
IC_17 Tumor_6: Basal cell like, keratins, EGFR
IC_18 Tumor_9_2: SLPI NDRG1 VEGFA, Lung9_2
IC_19 Tumor_5: Malignant Mesothelioma, CEACAM6 CXCL5 Lung5
IC_20 Myeloid: Macrophage
IC_21 Stroma: Type 2 alveolar
IC_22 Tumor_9: SLPI SOX4 FGFR1, common Lung9
IC_23 Lymphoid: B cells IgG
IC_24 Myeloid: Neutrophil
IC_25 Myeloid: Macrophage complement
IC_26 Stroma: Goblet cells
IC_27 Tumor_6: Keratins DST, Lung6
IC_28 Tumor_13: AREG MET Lung13
IC_29 Myeloid: M2 Macrophage
IC_30 Lymphoid: B cells IgM
IC_31 Cytokines: CXCL10
IC_32 Tumor_12,Tumor_13: SERPINA1, S100A2, THBS1 Lung12 13
IC_33 Tumor_5: epithelial progenitor MMP7 ITGB6 CEACAM6, Lung5
IC_34 Lymphoid: B cells IgM
IC_35 Stroma: Fibroblast TIMP1, Lung5
IC_36 Stroma: Airway smooth muscle
IC_37 Tumor_5,Tumor_6,Tumor_12: S100A6 S100P SLC40A1 KRT19, common tumor
IC_38 Lymphoid: MZB1 B cells IgG A
IC_39 Myeloid: Dendritic cell
IC_40 Stroma: Fibroblast MGP, Lung5
IC_41 Tumor_6: Basal cell like, keratins, EGFR
IC_42 Tumor_5,Tumor_9,Tumor_13: CEACAM6 MMP1 OLFM4, common tumor interface
IC_43 Stroma: Fibroblast
IC_44 : IL7R CCL19 CCL21
IC_45 Tumor_9: VEGFA PTGS2 SOX4, Lung9
IC_46 Tumor_9: CXCL1 CXCL3 SLPI, Lung9
IC_47 Lymphoid: T cells
IC_48 Myeloid: Macrophage CCL18
IC_49 Stroma: Endothelial cell
IC_50 :
IC_51 :
IC_52 Tumor_13: RPS4Y1 MET, OLFM4 neg, Lung13
IC_53 Stroma: Endothelial cell
IC_54 Tumor_9: SOX4 ALCAM DNMTs
IC_55 : Tiling artifact
IC_56 Stroma: Fibroblast THBS1
IC_57 Stroma: Fibroblast PTGDS
IC_58 Myeloid: Macrophage Complement CXCL9
IC_59 Tumor_5,Tumor_6,Tumor_12: KRT19 CEACAM6 ITGB4 EPAH2, common tumor
IC_60 Tumor: MSMB OLFM4
IC_61 Cytokines: CCL21
IC_62 Stroma: Pericytes
IC_63 Tumor_6: keratins, Lung6
IC_64 Tumor_12: PSCA PLAC8, common tumor
IC_65 Tumor_12: PIGR, Lung12
IC_66 Stroma: Smooth muscle cell
IC_67 Tumor_5: GPX3 VWF, Lung5
IC_68 Tumor_6: S100A2 Keratins, Lung6
IC_69 Stroma: Pulmonary alveolar
IC_70 Stroma: Myofibroblast IFN response
IC_71 Stroma: Fibroblast MT2A
IC_72 Tumor_9: SLPI CXCL10 MX1 OAS, Lung9
IC_73 Myeloid: Alveolar Macrophage
IC_74 Lymphoid: CD8 CTL or NK
IC_75 :
IC_76 :
IC_77 cell function: TNFa signalling, HSPs
IC_78 Tumor_6: CXCL14 keratins, Lung6
IC_79 Myeloid: Monocytes
IC_80 Stroma: fibroblast interface IGFBP3
IC_81 : IFN binding
IC_82 Stroma: type 2 Pneumocyte
IC_83 Myeloid: Monocytes
IC_84 Myeloid,Cytokines: CCL3 4 IL1B IL1RN CXCL8
IC_85 :
IC_86 :
IC_87 Tumor: Cycling, common tumor
IC_88 Stroma,Cytokines: CXCL2 CXCL3 ICAM1
IC_89 :
IC_90 Tumor_12,Tumor_13: LCN2 LYZ PIGR SERPINA1
IC_91 :
IC_92 Lymphoid: B cells IgD
IC_93 :
IC_94 :
IC_95 :
IC_96 :
IC_97 :
IC_98 :
IC_99 Stroma: Erythrocyte
IC_100 :
Lung_5_1
Lung_5_2
Lung_5_3
Lung_6
Lung_9_1
Lung_9_2
Lung_12
Lung_13
sample
