# Supplementary material for: CellsFromSpace: a fast, accurate, and reference-free tool to deconvolve and annotate spatially distributed omics data
Source: Bioinform Adv. 2024 May 30;4(1):vbae081. doi: 10.1093/bioadv/vbae081 (PMC11194756; doi:10.1093/bioadv/vbae081)
Supplement: vbae081_Supplementary_Data [file vbae081_supplementary_data.zip › SuppFigure4_MERSCOPE.pdf]

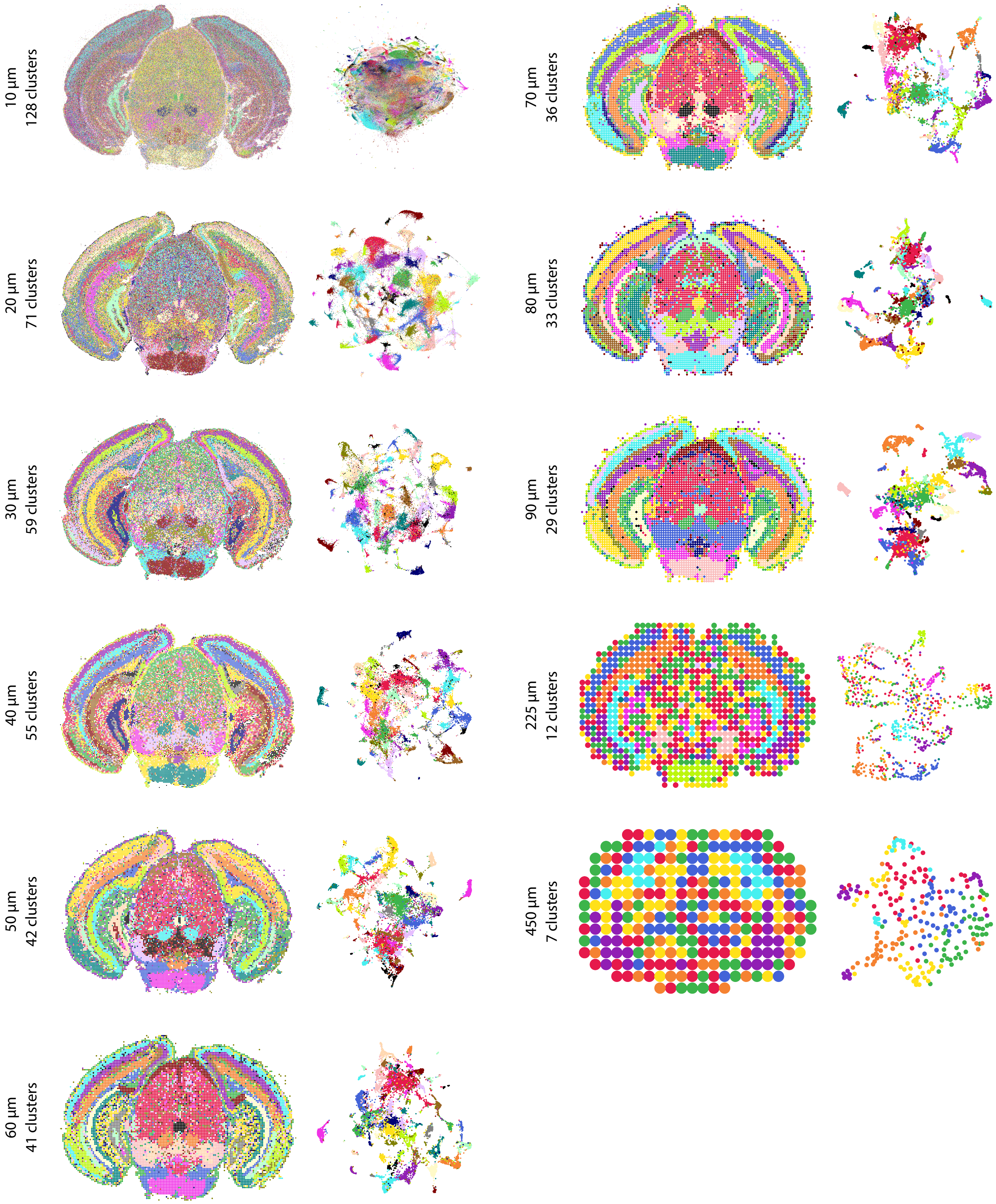

10 µm
128 clusters
20 µm
71 clusters
30 µm
59 clusters
40 µm
55 clusters
50 µm
42 clusters
60 µm
41 clusters
70 µm
36 clusters
80 µm
33 clusters
90 µm
29 clusters
225 µm
12 clusters
450 µm
7 clusters
