# Supplementary figures and images for: CellsFromSpace: a fast, accurate, and reference-free tool to deconvolve and annotate spatially distributed omics data
Source: Bioinform Adv. 2024 May 30;4(1):vbae081. doi: 10.1093/bioadv/vbae081 (PMC11194756; doi:10.1093/bioadv/vbae081)

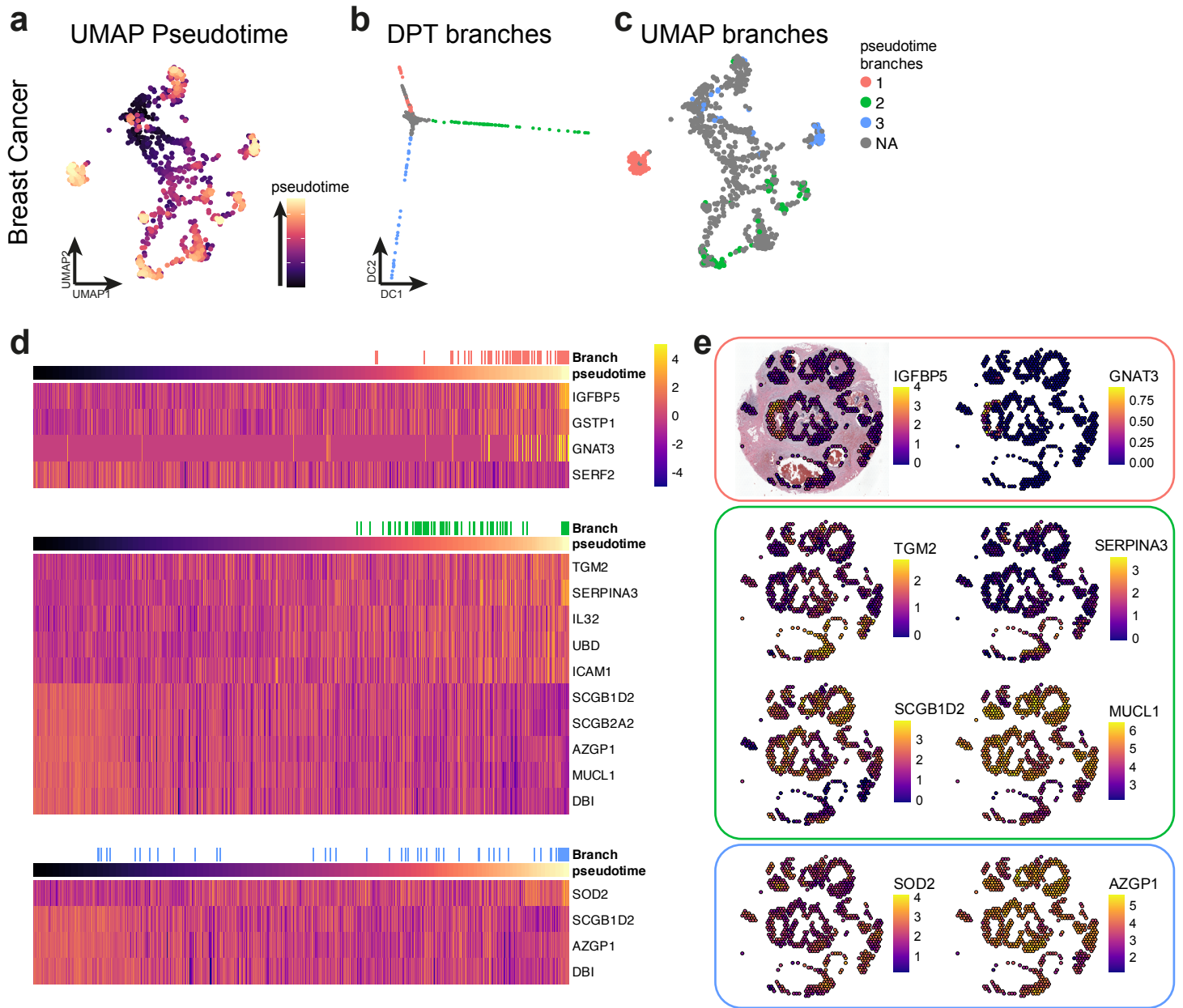

Supplement: vbae081_Supplementary_Data [file vbae081_supplementary_data.zip › SuppFigure2_trajectory.pdf]

**a mouse brain**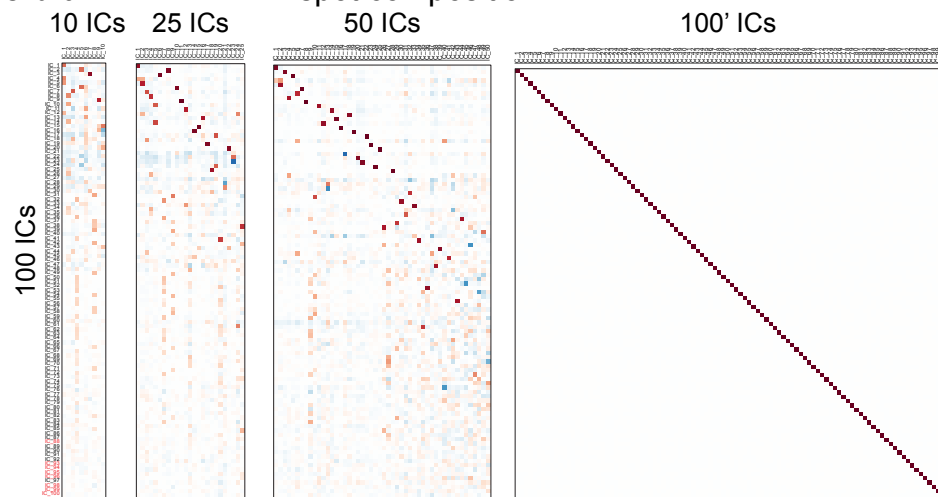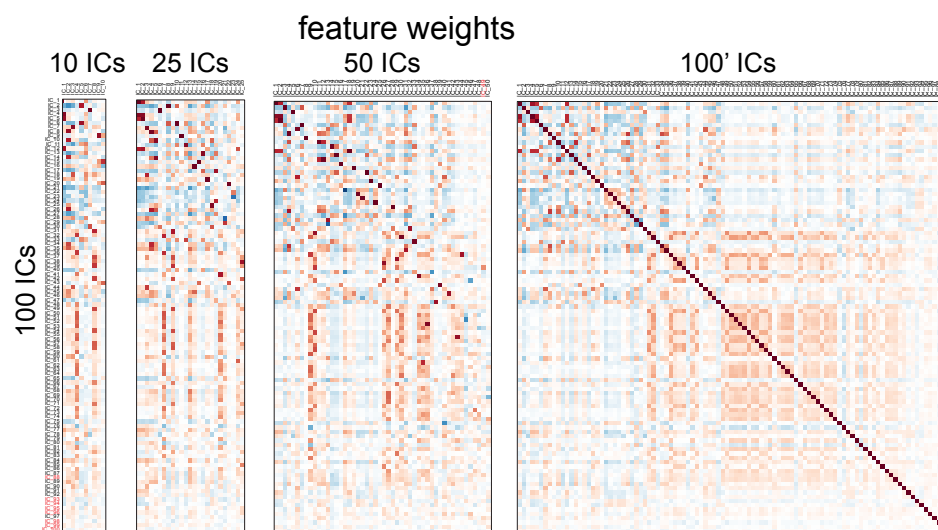**b human breast cancer**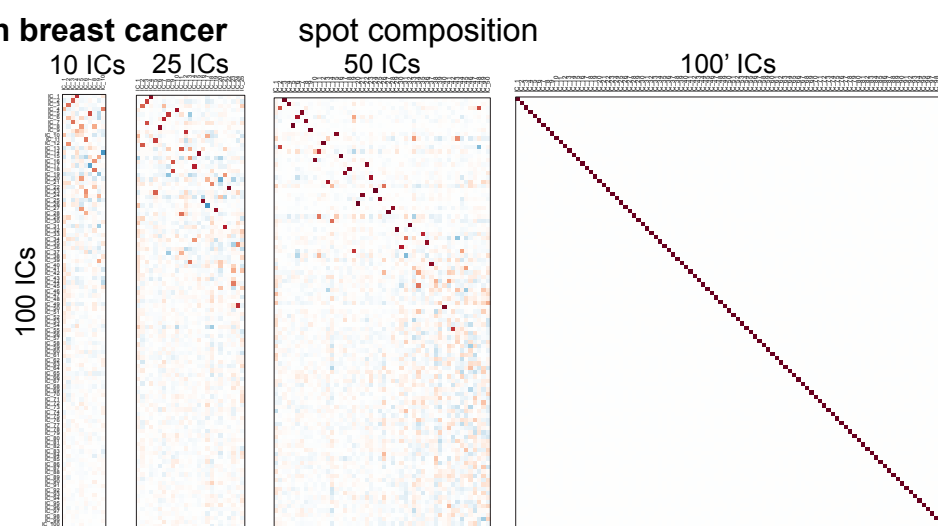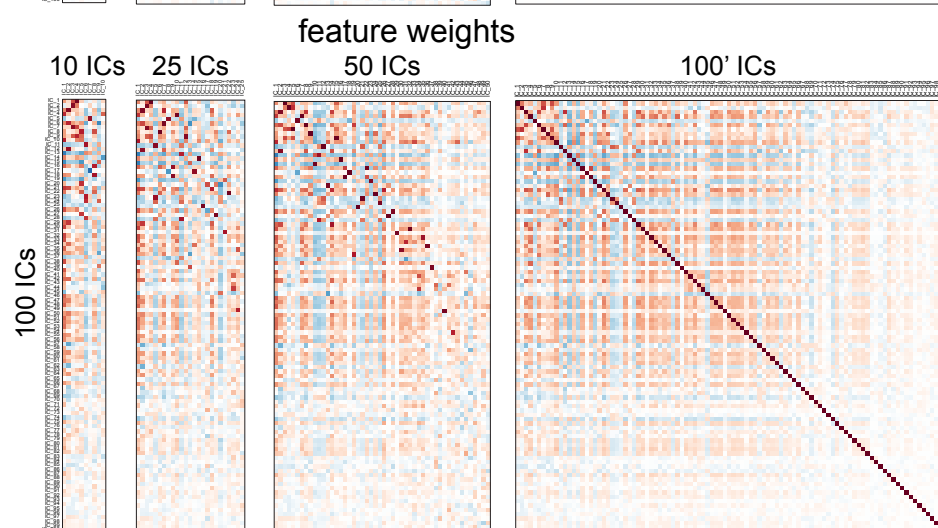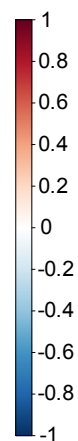

Supplement: vbae081_Supplementary_Data [file vbae081_supplementary_data.zip › SuppFigure10_impact_nICs.pdf]

# Mouse brain impact of # variable genes

all genes

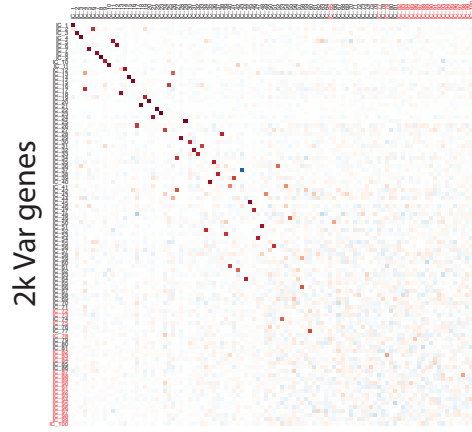

all genes

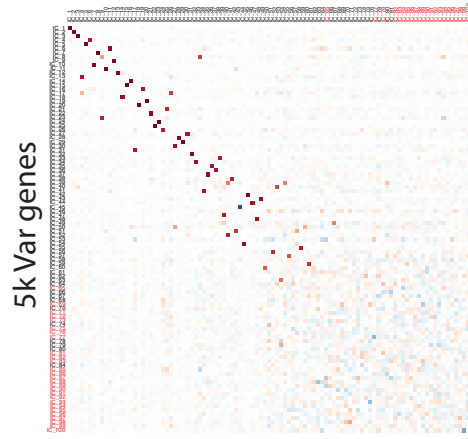

all genes iteration 1

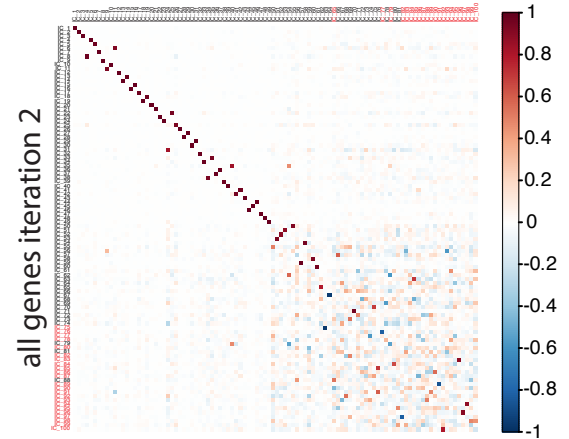

Supplement: vbae081_Supplementary_Data [file vbae081_supplementary_data.zip › SuppFigure9_impact_nVarGenes.pdf]

**a**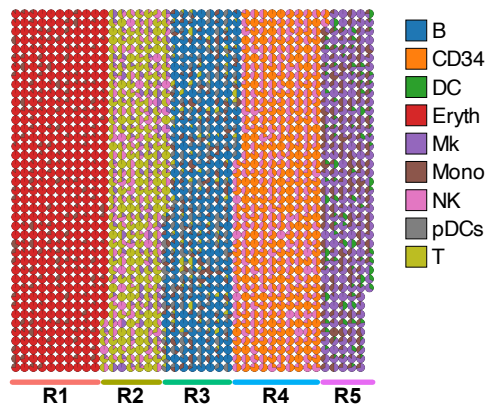**d**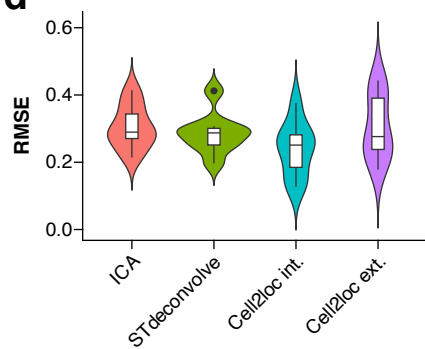**b Dimred**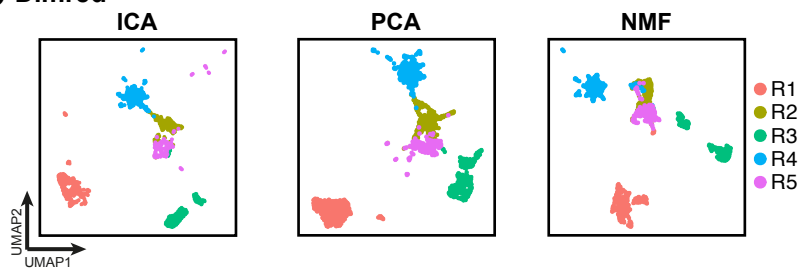**c Clustering**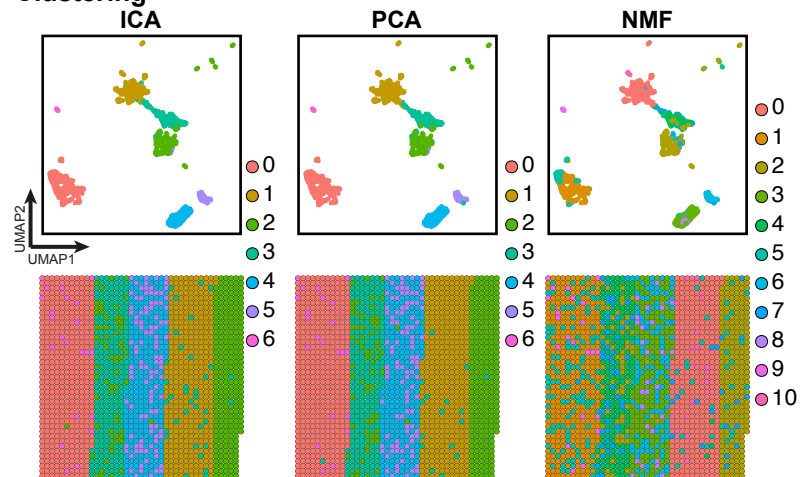**e**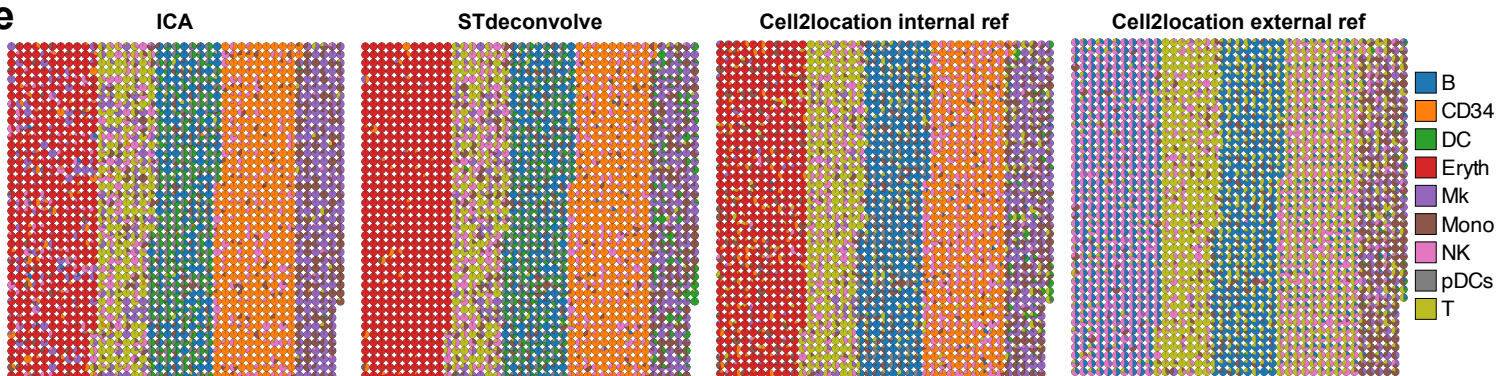

Supplement: vbae081_Supplementary_Data [file vbae081_supplementary_data.zip › SuppFigure11_synth_raster.pdf]

Ground truth vs CFS

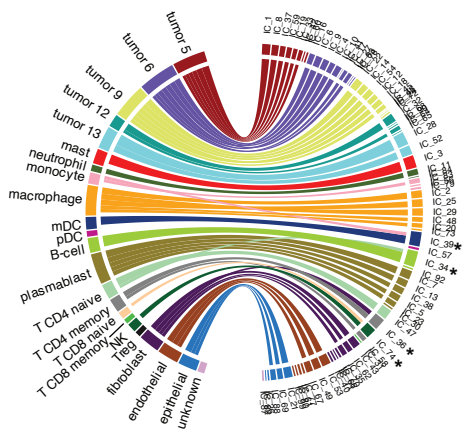

Ground truth vs STdeconvolve

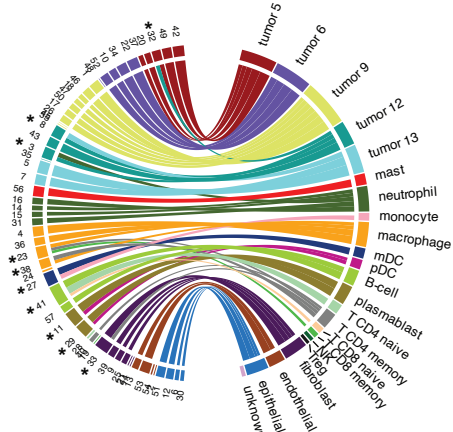

CFS vs STdeconvolve

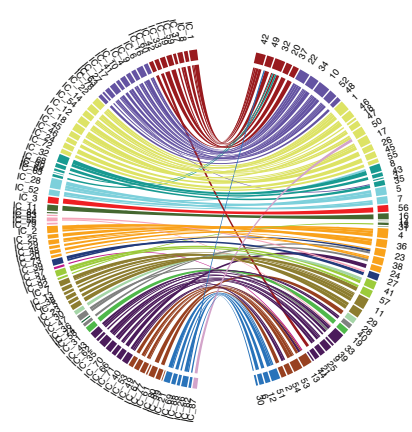

Supplement: vbae081_Supplementary_Data [file vbae081_supplementary_data.zip › suppFigure8_spotCompCorr.pdf]

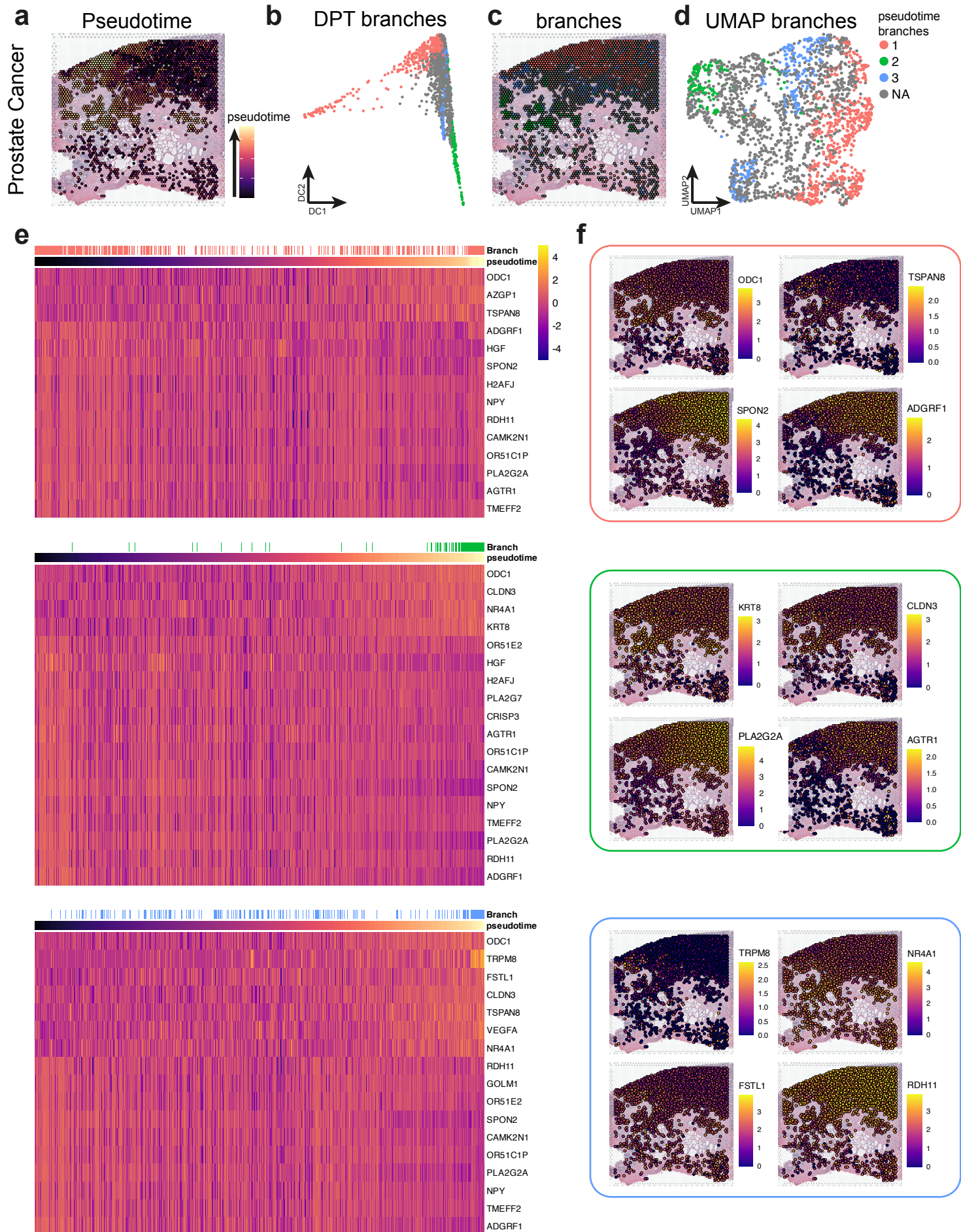

Supplement: vbae081_Supplementary_Data [file vbae081_supplementary_data.zip › suppFigure3_TrajectoryProstate.pdf]

**a**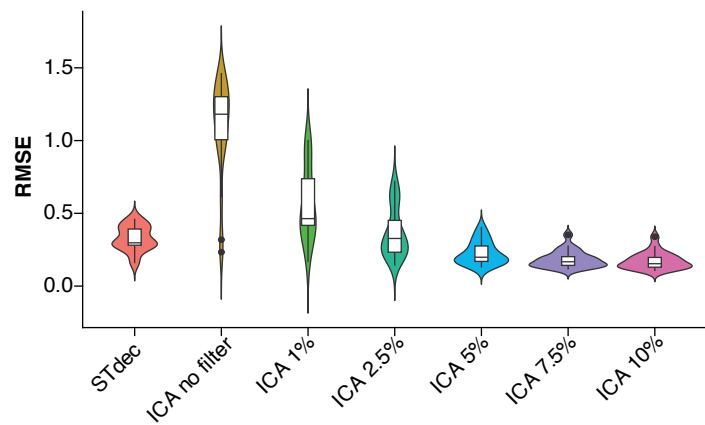**b**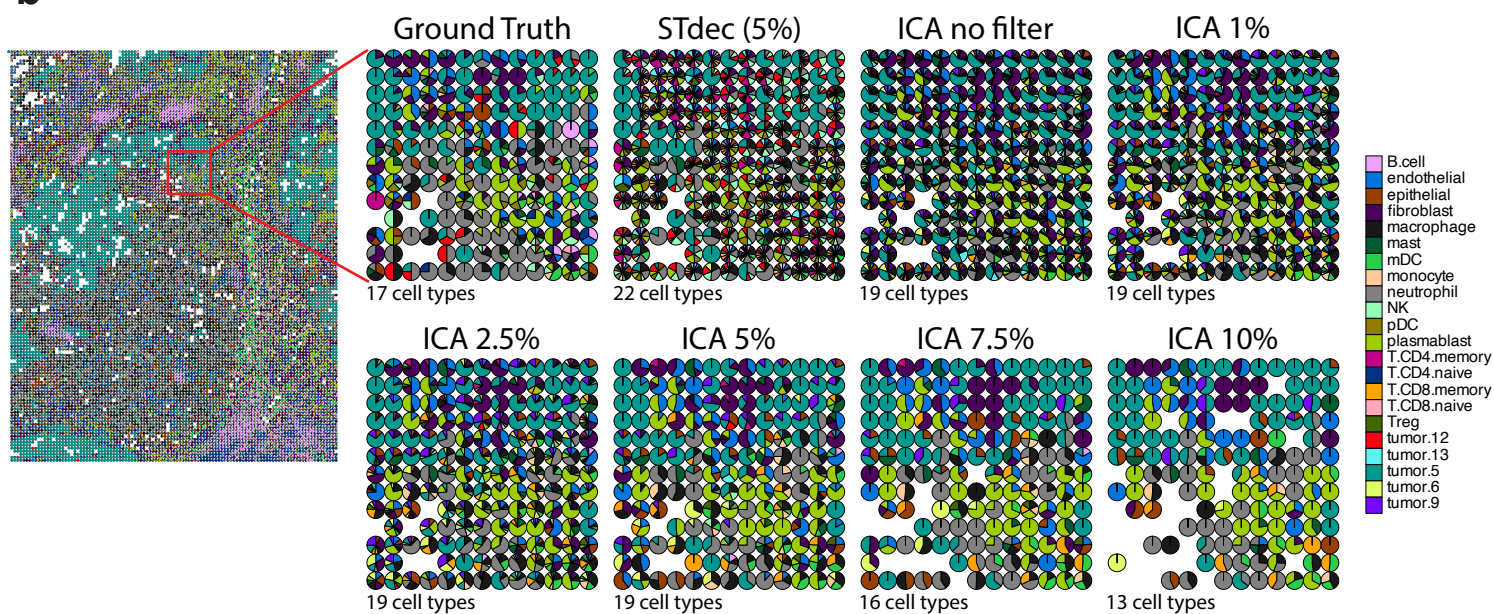

Supplement: vbae081_Supplementary_Data [file vbae081_supplementary_data.zip › SuppFigure12_ICAfilter.pdf]

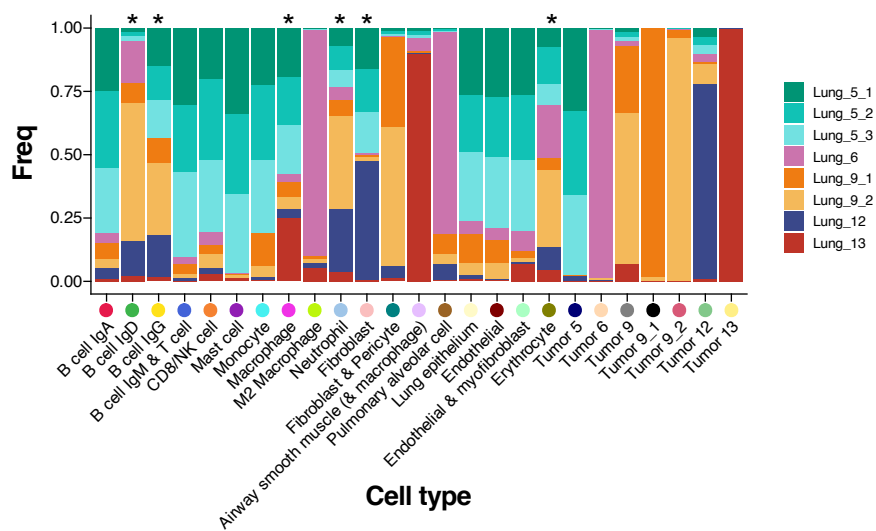

Supplement: vbae081_Supplementary_Data [file vbae081_supplementary_data.zip › SuppFigure6_CosMX_bySample.pdf]

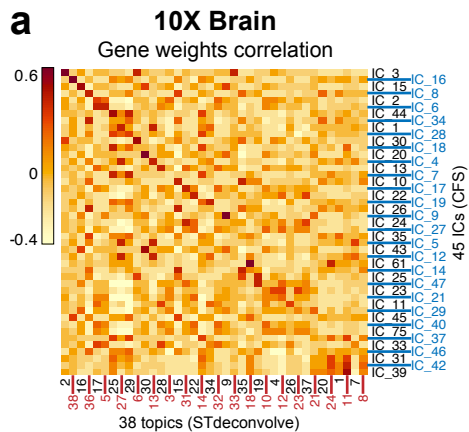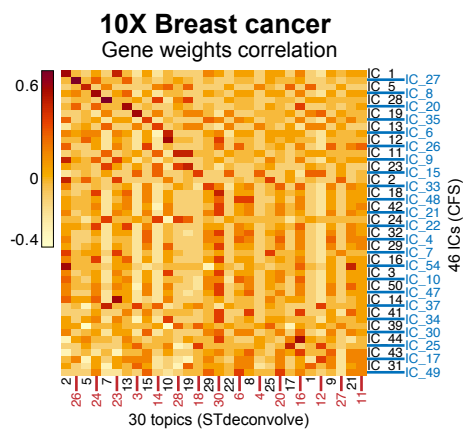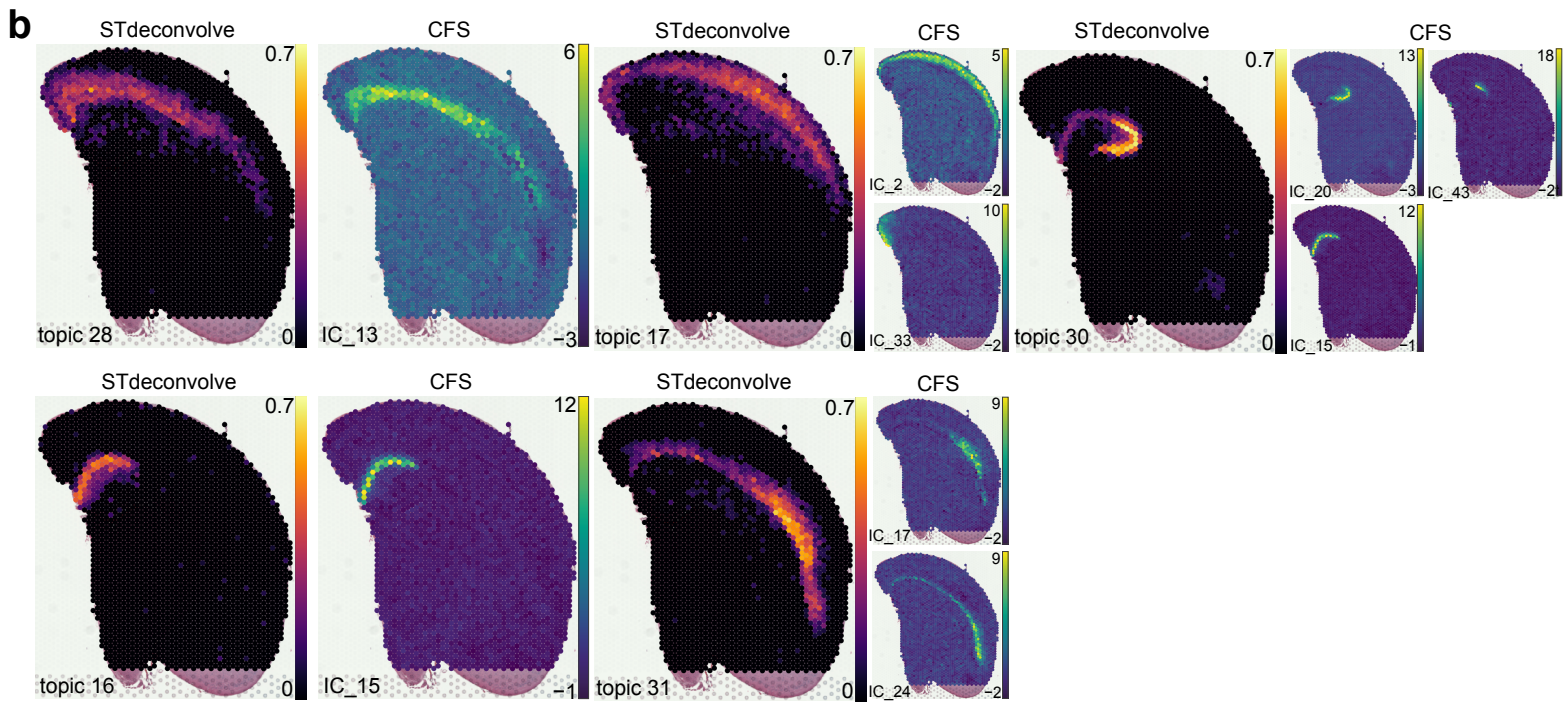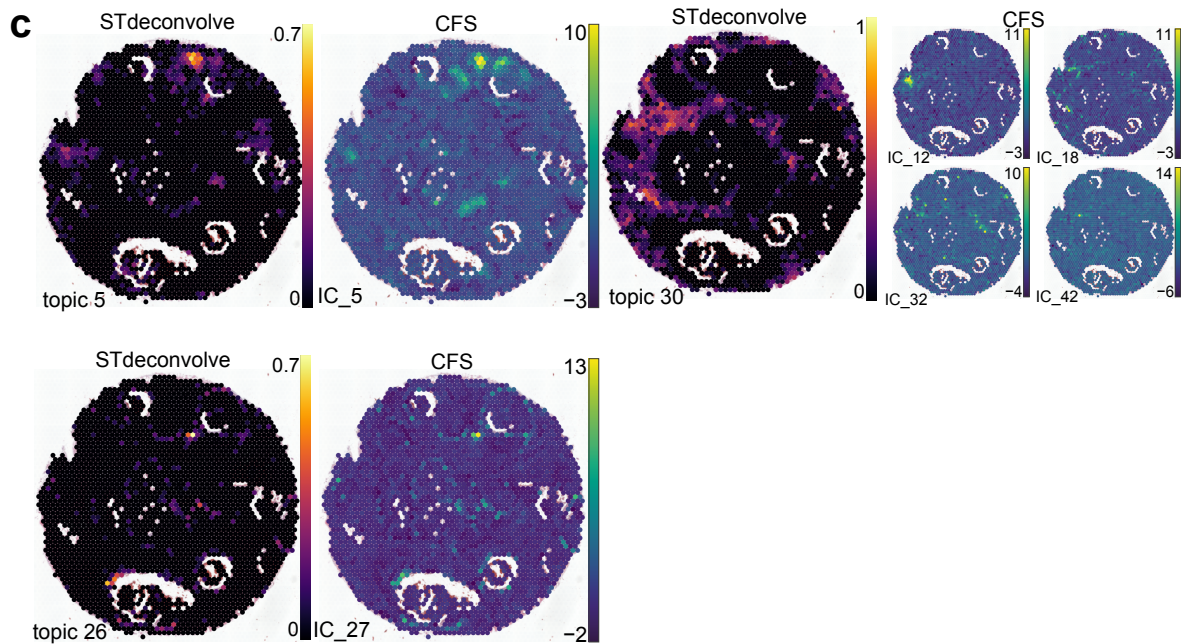

Supplement: vbae081_Supplementary_Data [file vbae081_supplementary_data.zip › suppFigure7_benchmark.pdf]

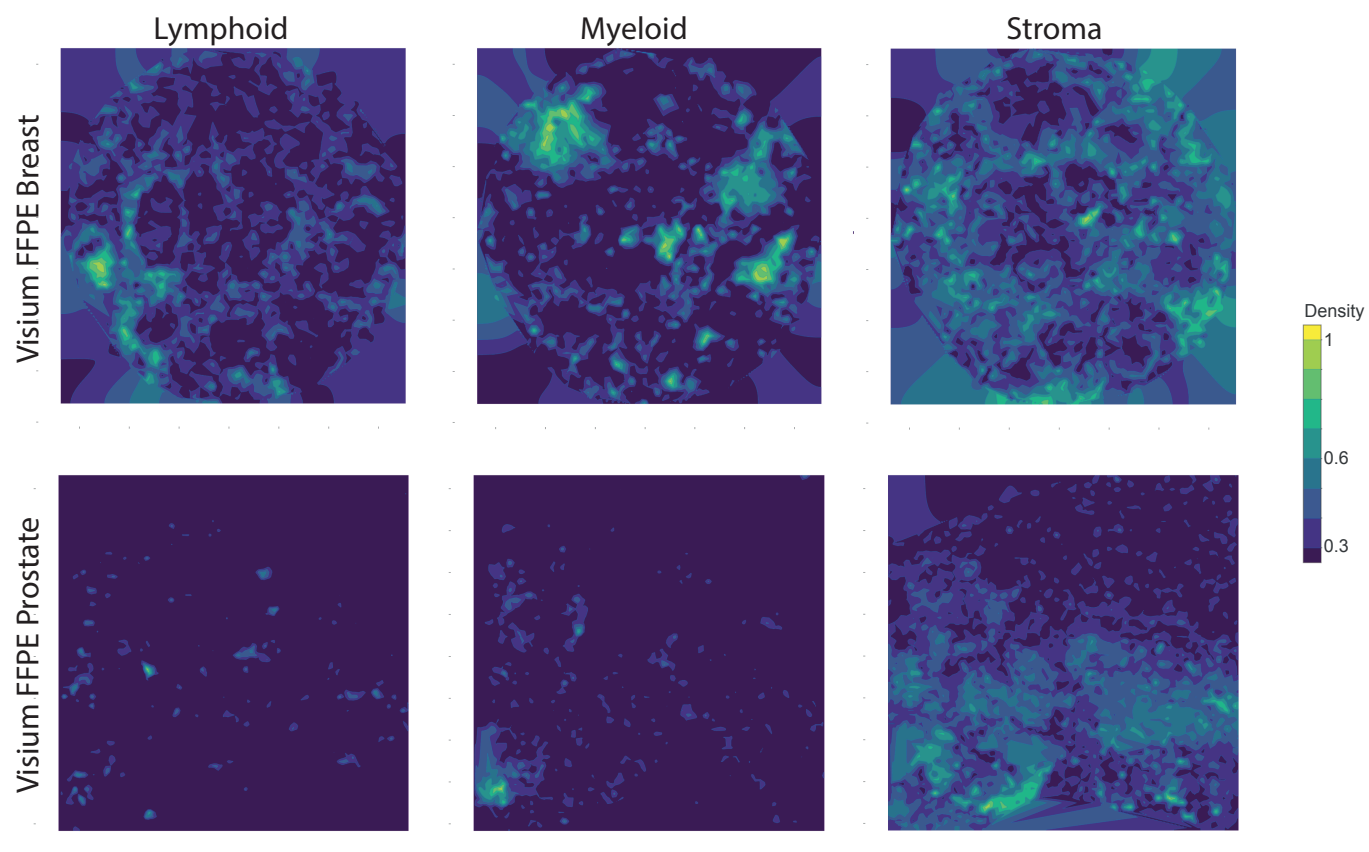

Supplement: vbae081_Supplementary_Data [file vbae081_supplementary_data.zip › SuppFigure1_Visium_Density.pdf]
